# Cross-Coupling Reaction of Allylic Ethers with Aryl Grignard Reagents Catalyzed by a Nickel Pincer Complex

**DOI:** 10.3390/molecules24122296

**Published:** 2019-06-21

**Authors:** Toru Hashimoto, Kei Funatsu, Atsufumi Ohtani, Erika Asano, Yoshitaka Yamaguchi

**Affiliations:** Department of Advanced Materials Chemistry, Graduate School of Engineering, Yokohama National University, 79-5 Tokiwadai, Hodogaya-ku, Yokohama 240-8501, Japan; funatsu-kei-rh@ynu.jp (K.F.); ohtani-atsufumi-cs@ynu.jp (A.O.); asano-erika-cy@ynu.jp (E.A.)

**Keywords:** pincer-type nickel(II) complex, cross-coupling, allylic ether

## Abstract

A cross-coupling reaction of allylic aryl ethers with arylmagnesium reagents was investigated using β-aminoketonato- and β-diketiminato-based pincer-type nickel(II) complexes as catalysts. An β-aminoketonato nickel(II) complex bearing a diphenylphosphino group as a third donor effectively catalyzed the reaction to afford the target cross-coupled products, allylbenzene derivatives, in high yield. The regioselective reaction of a variety of substituted cinnamyl ethers proceeded to give the corresponding linear products. In contrast, α- and γ-alkyl substituted allylic ethers afforded a mixture of the linear and branched products. These results indicated that the coupling reaction proceeded via a π-allyl nickel intermediate.

## 1. Introduction

Transition metal-catalyzed cross-coupling reactions are efficient and widely utilized synthetic protocols for constructing carbon–carbon bonds [1]. The reactions of allylic electrophiles with aryl metal reagents provide promising methodologies for the formation of C(sp^3^)–C(sp^2^) bonds. Allylic electrophiles with efficient leaving groups, such as allylic halides, acetates, and phosphates, are commonly used in these reactions [2,3,4,5,6]. In contrast to these allylic electrophiles, allylic ethers have been considered less-reactive electrophiles in cross-coupling reactions, whereas these electrophiles are easily accessible, cheap, and easy to handle. Therefore, the development of effective metal catalysts for coupling reactions utilizing allylic ethers as electrophiles in a highly regioselective manner has attracted considerable attention. The choice of the metal catalyst is critical for linear and branched selectivity. Although precious metals such as Pd and Ir have been extensively explored in selective allylic substitution reactions [2,3,4], recent efforts have focused on first-row late transition-metals such as Cu, Co, Fe, and Ni, whose complexes act as highly active catalysts. For example, Feringa reported the asymmetric allylic alkylation of acyclic allylic ethers with organolithium reagents using a Cu/phosphoramidite system [7]. Oshima and Yorimitsu reported a cobalt-catalyzed cross-coupling reaction of allylic ethers with Grignard reagents in a linear manner [8]. Iron catalysts also enable the allylic arylation reaction to be performed [9,10,11,12]. Recently, Ni pincer complexes have been reported as effective catalysts for the coupling reaction of allylic ethers with aryl zinc reagents [13].

**Scheme 1:**
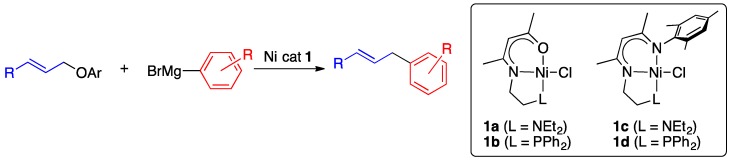
The coupling reaction of allylic ethers with aryl Grignard reagents using Ni(II) pincer complexes (**1a**–**d**).

In recent years, tridentate pincer-type complexes have generated significant interest because the pincer-type ligand stabilizes the metal complex and its properties can be tuned to achieve optimal reactivity [14,15,16,17,18,19,20,21,22,23,24,25]. We have recently reported the synthesis of a range of pincer-type nickel(II) complexes (**1a**–**d**) utilizing a combination of β-aminoketonato or β-diketiminato frameworks with a third donor such as an amino or phosphino group [26,27]. Our systematic study on these nickel(II) complexes revealed that modifying the ligand framework has a significant influence on the catalytic performance in the cross-coupling reaction of aryl halides with arylmagnesium reagents. In particular, nickel(II) complexes **1b** and **1d** bearing a diphenylphosphino group as the third donor enable the utilization of aryl fluorides as electrophiles in the reaction [27]. In order to elucidate the reactivity of the nickel(II) complex toward the relatively robust C-O bond in an allylic aryl ether, we herein describe the cross-coupling reaction of allylic ethers with arylmagnesium reagents using nickel(II) complexes (**1a**–**d**) (Scheme 1).

## 2. Results and Discussion

### 2.1. Optimization of the Reaction Conditions

#### 2.1.1. Initial Optimization of the Nickel(II) Complexes

To evaluate the catalytic activity of nickel(II) complexes **1a**–**d**, we carried out the reaction between cinnamyl phenyl ether **2a** and 3 equivalents of *p*-tolylmagnesium bromide **3a** using nickel(II) complexes **1a**–**d** (2.5 mol%) in THF at room temperature (Table 1), referring to our previously study on the biaryl coupling reaction of aryl chlorides with arylmagnesium reagents [26]. Complex **1a** gave the linear product **4a** in only 7% yield. Complex **1b** showed high catalytic activity to give **4a** in 86% yield. Nickel(II) complexes **1c** and **1d** bearing *N*,*N*,*N*- and *N*,*N*,*P*-type ligands were not effective in the reaction and afforded the target product in 2% and 4% yield, respectively. In these cases, a large amount of **2a** remained unreacted (see 2.3 Reaction mechanism section). In all cases, the branched product **5a** was not detected and 4,4′-bitolyl (**6a**) derived from **3a** was formed as a by-product (9–30%). In the absence of complex **1a**–**d**, the reaction did not give any coupled products. 

#### 2.1.2. Investigation of the Effect of the Solvent and Nickel(II) Complexes

We next examined the solvent effect using the reaction between **2a** and 3 equivalents of **3a** with complex **1b**. The results are summarized in Table 2. The choice of the solvent used is essential to produce product **4a** in satisfactory yield. When cyclopentyl methyl ether (CPME) was employed as solvent, the coupled product **4a** was obtained in 89% yield (entry 2). The yield of **4a** decreased in 1,4-dioxane (71%, entry 3). *t*-Butyl methyl ether (MTBE) and toluene were also effective solvents for the reaction and produced **4a** in 82% and 83%, respectively (entries 4 and 5). CH_2_Cl_2_ and *N*,*N*-dimethylformamide (DMF) were not suitable for the reaction (entries 6 and 7). Et_2_O provided the best result, affording product **4a** in 99% yield, from which **4a** was isolated in 94% yield (entry 8). The other nickel(II) complexes **1a**, **1c**, and **1d** exhibited relatively low catalytic activity, even in Et_2_O (entries 9–11). When the amount of Grignard reagent **3a** used was reduced to 1.5 equivalents, complex **1b** acted as an effective catalyst to give **4a** in an isolated yield of 91% (entry 12).

#### 2.1.3. Investigation of the Effect of the Leaving Group on the Allylic Electrophile

In order to investigate the influence of the leaving group (LG) on the allylic substrate, we carried out the reaction between various cinnamyl electrophiles (**2a**–**d**, **7**, and **8**) with 1.5 equivalents of **3a** in the presence of **1b** (2.5 mol%) in Et_2_O (Table 3). Cinnamyl phenyl ether **2a** was converted into **4a** in 91% isolated yield. In the case of cinnamyl *p*-methoxyphenyl ether **2b**, the cross-coupled product **4a** was isolated in excellent yield (96%). When the amount of nickel(II) complex **1b** used was reduced to 1 mol%, **2a** was converted into **4a** in 60% yield, whereas in the case of **2b**, product **4a** was isolated in 95% yield. The reaction of cinnamyl ether **2c** bearing a *p*-trifluoromethylphenyl group in the leaving group afforded **4a** in 62% yield. *p*-Methoxyphenoxide, which has an electron-donating group on the aromatic ring, acted as an effective leaving group. Methyl ether **2d** was also applicable in the reaction using 2.5 mol% of **1b** to give product **4a** in 90% yield. When 1 mol% of **1b** was employed, the product was obtained in 73% yield. Cinnamyl alcohol **7** was also able to be used as an electrophile, however, the yield of **4a** was low (40%) and **7** was recovered in 54%. In the case of cinnamyl chloride **8**, compound **4a** was formed in low yield (38%). 

### 2.2. Substrate Scope

With the optimized conditions in hand, we next examined the substrate scope of the reaction using allylic ethers **2** bearing a *p*-methoxyphenoxide group as the leaving group and a range of arylmagnesium reagents (**3**) in the presence of 1 mol% of **1b** as the catalyst (Table 4). The reaction of cinnamyl ether **2b** with phenylmagnesium bromide **3b** gave the coupled product **4b** in excellent yield (94%) with linear selectivity. In the case of 4-methoxyphenylmagnesium bromide **3c**, which has an electron-donating group on the aromatic ring, the coupled product **4c** was isolated in 94% yield. The reaction with 4-*N*,*N*-dimethylaminophenylmagnesium bromide **3d** gave product **4d** in 95% yield. We recently reported that complexes **1b** and **1d** act as an effective catalyst for the cross-coupling reaction of aryl fluorides with arylmagnesium reagents [27]. In the reaction with 4-fluorophenylmagnesium bromide **3e**, product **4e** was obtained in 96% yield. In the present reaction conditions, the reaction of **4e** as an electrophile with Grignard reagent **3e** did not occur. In cases of sterically congested arylmagnesium reagents, such as 1-naphthylmagnesium bromide **3f**, *o*-tolylmagnesium bromide **3g** and 2,4,6-trimethylphenylmagnesium bromide **3h**, the corresponding products **4f**, **4g**, and **4h** were obtained in 85%, 79%, and 80%, respectively, in the presence of 2.5 mol% of **1b** under refluxing conditions. The reaction of *p*-substituted cinnamyl ether **2i**, which has an electron-donating group (OMe) on the aromatic ring, with *p*-tolylmagnesium bromide **3a** gave product **4i** in 78% yield. In the case of electrophile **2j** bearing an electron-withdrawing group (F), the coupled product **4j** was obtained in 89% yield. In the coupling reaction, alkyl- and benzylmagnesium reagents were not suitable as nucleophiles. In addition, the reaction using a *cis*/*trans* mixture of **2b′** (*cis*:*trans* = 86:14) with Grignard reagent **3a** afforded the *trans* product (**4a**) in 88% yield as a sole product (Table 5, entry 1). In contrast to cinnamyl ethers, γ-alkyl substituted allylic ether **2g** afforded a mixture of the linear and branched isomers (**4k** and **5k**) in 96% yield. The ratio of **4k**:**5k** was determined to be 54:46 using ^1^H NMR spectroscopy (entry 2). These results indicate that the reaction proceeds via a π-allyl nickel intermediate (vide infra). Therefore, we examined the reaction using secondary allylic ethers as electrophiles. The reaction of an allylic ether bearing a phenyl group at the α-position (**2h**) proceeded with linear selectivity to afford product **4a** as a single regioisomer in high yield (94%). In this reaction, branched isomer **5a** was not detected (entry 3). In contrast, an allylic ether bearing an alkyl group at the α-position (**2i**) was converted into a mixture of regioisomers (**4k** and **5k**) in 93% yield. The ratio of **4k**:**5k** was determined to be 57:43 using ^1^H NMR spectroscopy (entry 4). The reaction of a mixture of **2j** and **2k** (**2j**:**2k** = 67:33) gave the linear product **4l** in 90% yield with high regioselectivity. The branched isomer (**5l**) was not detected (entry 5). The regioselectivity in this reaction probably depends on both maximizing the resonance stabilization energy on the coupling product and minimizing steric hindrance in the coupling of the Grignard reagent with the putative π-allyl nickel species [13]. In the reaction of the γ-alkyl substituted allylic ether, the steric influence should operate predominantly for the formation of the product.

**Scheme 2:**
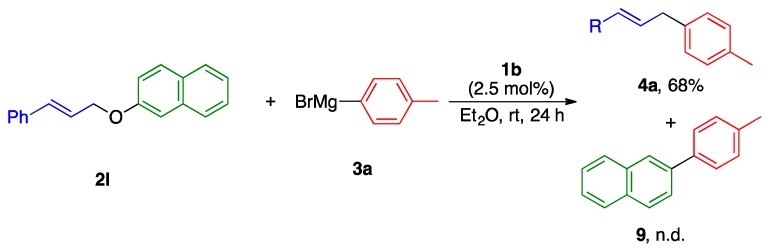
Selective cleavage of the allylic C(sp^3^)–O bond in cinnamyl naphthyl ether **2l** catalyzed by **1b**.

Finally, we investigated the reactivity of the allylic C(sp^3^)–O and naphthyl C(sp^2^)–O bonds in **2l** (Scheme 2). Scission of the allylic C(sp^3^)–O bond in **2l** selectively occurred and thus, allylated product **4a** was obtained in 68% yield. 

### 2.3. Reaction Mechanism

We examined the stoichiometric reaction to gain insight into the reaction mechanism (Table 6). In the reaction of nickel(II) complex **1b** with an equimolar amount of allylic ether **2b**, oxidative addition of the C-O bond of **2b** to **1b** did not occur and **2b** was recovered in 96%. In contrast, when **1b** was treated with an excess amount of phenylmagnesium reagent **3b**, **1b** was converted into another nickel species. In the ^31^P{^1^H} NMR spectrum, the signal derived from **1b** (37.5 ppm) disappeared [26] and a new signal was observed at 49.1 ppm, whose resonance was due to the formation of a new nickel species. However, characterization of this nickel complex was not successful. Subsequently, we examined the reaction with various amounts of **3a** in the presence of **1b** and **2b**. The obtained results are summarized in Table 6. In the reaction using a mixture of **1b** and **2b** with 1 equivalent of **3a**, the coupled product **4a** was not detected with **2b** being recovered in 83%. In the case of the reaction using 2 equivalents of **3a**, the coupled product (**4a**) was formed in 23% yield. Using 3 equivalents of **3a**, the reaction proceeded smoothly and **2b** was completely consumed to give the coupled product (**4a**) in 83% yield, along with the formation of 4,4′-bitolyl (**6a**, 0.19 mmol). The reaction using 4 equivalents of **3a** also gave the product (**4a**) in 83% yield. Therefore, we assumed that two or more equivalents of the arylmagnesium reagent are required to for the reaction to proceed via the generation of an anionic nickel(0) species, which is the active species for the activation of the allylic ether (vide infra). 

**Scheme 3:**
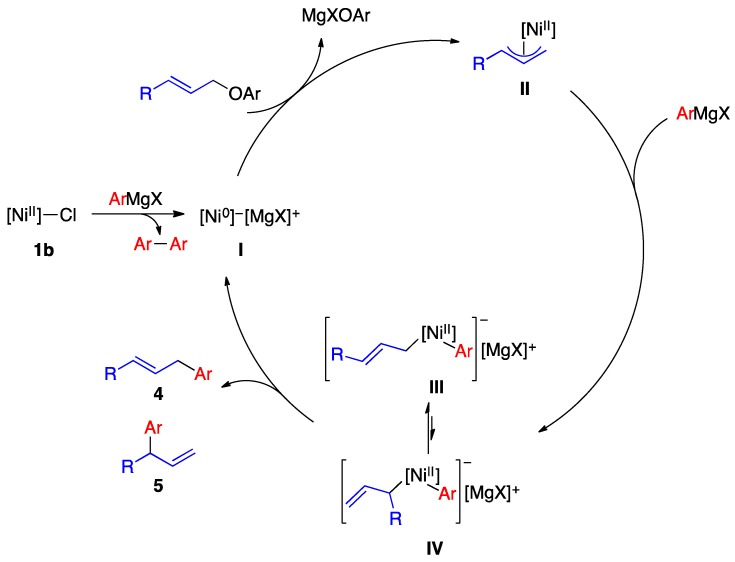
A plausible catalytic cycle for the cross-coupling reaction of an allylic ether and arylmagnesium reagent.

Although the detailed reaction mechanism for the nickel catalyzed allyl-aryl coupling reaction was not clear, a plausible mechanism is proposed in Scheme 3 [13,28]. Initially, nickel(II) complex **1b** is converted into anionic nickel(0) species **I** through its reaction with at least 2 equivalents of arylmagnesium reagent accompanied by the formation of a biaryl. Then, the low valent nickel(0) species (**I**) reacts with the allylic electrophile at the nickel center to afford π-allyl nickel species **II** (as shown in Table 5): For example, γ-alkyl substituted allylic ether **2g** was converted into a mixture of linear and branched isomers. We have recently reported that the steric and electronic properties of the nickel(II) complexes **1** [26,27]. The DFT calculations of complexes **1** reveal that the highest occupied molecular orbital (HOMO) levels of these complexes increase in the order **1a** ≈ **1b** < **1c** ≈ **1d**. We expected that complexes **1c** and **1d** worked as effective catalysts for this coupling reaction. However, the bulky substituent (mesityl group) on the ligand framework inhibits to form π-allyl nickel species derived from **1c** and **1d**. Therefore, complexes **1c** and **1d** show poor catalytic activities. In contrast, complex **1b** acts as an effective catalyst for the coupling reaction, because of constructing an appropriate steric and electronic environment around nickel center by the *O,N,P*-pincer ligand. After the formation of π-allyl nickel species **II**, its subsequent transmetalation with the arylmagnesium reagent leads to the formation of a σ-allyl nickel species bearing aryl ligand **III** and/or **IV**. We assumed that the formation of intermediate **III** is more favorable than **IV** due to steric hindrance. Therefore, the selectivity in the reaction was influenced by the substituent on the allylic substrate. Finally, the desired products **4** and/or **5** are released via reductive elimination from the Ni center to regenerate the nickel(0) species (**I**) and thus, the catalytic cycle is completed. One of the possible reaction pathways involved in an allylic substitution reaction is the attack of the nucleophile on π-allyl nickel species **I**; this mechanism cannot be excluded. 

## 3. Materials and Methods

### 3.1. General Information

All manipulations involving air- and moisture-sensitive organometallic compounds were carried out under a nitrogen atmosphere, which was dried with SICAPENT (Merck Co., Inc., Germany, Darmstadt), using standard Schlenk tube or high vacuum techniques. All solvents were distilled over appropriate drying agents prior to use. Nickel complexes **1a**–**c** [26] and **1d** [27] were prepared according to literature methods. Allylic aryl ethers **2a**–**c**, **2e**–**g**, and **2l** were prepared from the reaction of their corresponding allylic halides and 4-substituted phenols using K_2_CO_3_ in acetone. Allylic methyl ether **2d** was prepared via the reduction of the corresponding aldehyde using NaBH_4_ followed by methylation using methyl iodide. Allylic methyl ethers **2h** and **2i** were prepared from addition of the corresponding aldehyde with vinylmagnesium bromide followed by methylation using methyl iodide. A mixture of **2j** and **2k** was prepared via the reduction of cinnamyl methyl ketone using NaBH_4_ followed by a Mitsunobu reaction using 4-methoxyphenol. All other reagents employed in this research were commercially available and used without any further purification.

Proton nuclear magnetic resonance (^1^H NMR), carbon nuclear magnetic resonance (^13^C{^1^H} NMR), and phosphorus nuclear magnetic resonance (^31^P{^1^H} NMR) spectra were recorded on BRUKER DRX-500 and JEOL ECA 500 spectrometers at ambient temperature. ^1^H NMR chemical shifts were recorded in ppm using tetramethylsilane (TMS) or the solvent resonance peak as an internal standard (TMS: 0.00 ppm, C_6_D_6_: 7.16 ppm). ^13^C{^1^H} NMR chemical shifts were recorded in ppm using the solvent resonance peak as an internal standard (CDCl_3_: 77.0 ppm, C_6_D_6_: 128.0 pm). ^31^P{^1^H} NMR chemical shifts were recorded in ppm using H_3_PO_4_ as an external standard (H_3_PO_4_: 0.0 ppm).

### 3.2. General Procedure for the Cross-Coupling Reaction of Allylic Ethers with Arylmagnesium Reagents 

A 50 mL Schlenk tube was charged with **1b** (1.0–2.5 mol%), the respective allylic electrophile (1.0 mmol), Et_2_O (2.5–5 mL, 0.2 M based on the electrophile), and the aryl Grignard reagent (1.5–3.0 equiv.) at room temperature. The coupling reaction was carried out at room temperature for 24 h. After quenching with 1 M HCl aq. (5 mL), the aqueous layer was extracted with EtOAc (5 × 3 mL). The combined organic layers were washed with brine (10 mL) and dried over anhydrous MgSO_4_. After filtration and removal of all volatiles from the filtrate, the residue was purified by column chromatography on silica gel. 

*1-Cinnamyl-4-methylbenzene* (**4a**): The compound **4a** was synthesized from 1-methoxy-4-{[(*2E*)-3-phenylprop-2-en-1-yl]oxy}benzene **2b** (240.9 mg, 1.00 mmol) with *p*-tolylmagnesium bromide **3a** (1.4 mL, 1.1 M in THF, 1.5 mmol) in the presence of **1b** (4.1 mg, 1 mol%) in Et_2_O (5 mL) at room temperature for 24 h (198.0 mg, 95% yield, colorless liquid). ^1^H and ^13^C{^1^H} NMR spectra have been attached in the Supplementary Materials. The product was characterized by comparison with the previously reported ^1^H and ^13^C{^1^H} NMR data [29].

The compound **4a** was synthesized from **2b′** (*cis*:*trans* = 86:14, 119.6 mg, 0.50 mmol) with **3a** (0.70 mL, 1.1 M in THF, 0.77 mmol) in the presence of **1b** (2.0 mg, 1 mol%) in Et_2_O (2.5 mL) at room temperature for 24 h (91.1 mg, 88% yield).

The compound **4a** was synthesized from (1-methoxy-2-propen-1-yl)benzene **2h** (147.3 mg, 0.99 mmol) with **3a** (1.40 mL, 1.1 M in THF, 1.5 mmol) in the presence of **1b** (10.1 mg, 2.5 mol%) in Et_2_O (5 mL) at room temperature for 24 h (194.2 mg, 94% yield).

The compound **4a** was synthesized from 2-{[(2*E*)-3-phenylprop-2-en-1-yl]oxy}naphthalene **2l** (260.9 mg, 1.00 mmol) with **3a** (1.4 mL, 1.09 M in THF, 1.5 mmol) in the presence of **1b** (10.1 mg, 2.5 mol%) in Et_2_O (5 mL) at room temperature for 24 h. The yield of **4a** was determined by ^1^H NMR analysis of the crude product using pyrazine as the internal standard (68% yield).

*(E)-Prop-1-ene-1,3-diyldibenzene* (**4b**). The compound **4b** was synthesized from **2b** (238.8 mg, 0.99 mmol) with phenylmagnesium bromide **3b** (1.4 mL, 1.1 M in THF, 1.5 mmol) in the presence of **1b** (4.06 mg, 1 mol%) in Et_2_O (5 mL) at room temperature for 24 h (181.2 mg, 94% yield, colorless liquid). ^1^H and ^13^C{^1^H} NMR spectra have been attached in the Supplementary Materials. The product was characterized by comparison with the previously reported ^1^H and ^13^C{^1^H} NMR data [30].

1-Cinnamyl-4-methoxylbenzene (**4c**). The compound **4c** was synthesized from **2b** (238.4 mg, 0.99 mmol) with 4-methoxylphenylmagnesium bromide **3c** (3.0 mL, 0.5 M in THF, 1.5 mmol) in the presence of **1b** (3.90 mg, 1 mol%) in Et_2_O (5 mL) at room temperature for 24 h (209.1 mg, 94% yield, colorless liquid). ^1^H and ^13^C{^1^H} NMR spectra have been attached in the Supplementary Materials. The product was characterized by comparison with the previously reported ^1^H and ^13^C{^1^H} NMR data [29].

*1-Cinnamyl-N,N-dimethylaniline* (**4d**). The compound **4d** was synthesized from **2b** (239.2 mg, 1.00 mmol) with 4-*N*,*N*-dimethylaminophenylmagnesium bromide **3d** (2.0 mL, 0.75 M in THF, 1.5 mmol) in the presence of **1b** (10.2 mg, 2.5 mol%) in Et_2_O (5 mL) at room temperature for 24 h (224.5 mg, 95% yield, yellow liquid). ^1^H and ^13^C{^1^H} NMR spectra have been attached in the Supplementary Materials. The product was characterized by comparison with the previously reported ^1^H and ^13^C{^1^H} NMR data [29].

*1-Cinnamyl-4-fluorobenzene* (**4e**). The compound **4e** was synthesized from **2b** (241.1 mg, 1.00 mmol) with 4-fluorophenylmagnesium bromide **3e** (1.5 mL, 1.0 M in THF, 1.5 mmol) in the presence of **1b** (9.9 mg, 2.5 mol%) in Et_2_O (5 mL) at room temperature for 24 h (230.1 mg, 96% yield, colorless liquid). ^1^H and ^13^C{^1^H} NMR spectra have been attached in the Supplementary Materials. The product was characterized by comparison with the previously reported ^1^H and ^13^C{^1^H} NMR data [29].

*1-Cinnamylnaphthalene* (**4f**). The compound **4f** was synthesized from **2b** (122.0 mg, 0.51 mmol) with 1-napthylmagnesium bromide **3f** (5.8 mL, 0.26 M in THF, 1.51 mmol) in the presence of **1b** (5.1 mg, 2.5 mol%) in Et_2_O (5 mL) at reflux conditions for 72 h (105.5 mg, 85% yield, white solid). ^1^H and ^13^C{^1^H} NMR spectra have been attached in the Supplementary Materials. The product was characterized by comparison with the previously reported ^1^H and ^13^C{^1^H} NMR data [31].

*1-Cinnamyl-2-methylbenzene* (**4g**). The compound **4g** was synthesized from **2b** (240.3 mg, 1.00 mmol) with *o*-tolyl magnesium bromide **3g** (3.5 mL, 0.63 M in THF, 2.2 mmol) in the presence of **1b** (10.3 mg, 2.5 mol%) in Et_2_O (5 mL) at reflux conditions for 24 h (164.7 mg, 79% yield, colorless liquid). ^1^H and ^13^C{^1^H} NMR spectra have been attached in the Supplementary Materials. The product was characterized by comparison with the previously reported ^1^H and ^13^C{^1^H} NMR data [29].

*1-Cinnamyl-2,4,6-trimethylbenzene* (**4h**). The compound **4h** was synthesized from **2b** (241.7 mg, 1.01 mmol) with 2,4,6-trimethylphenyl magnesium bromide **3h** (3.5 mL, 0.86 M in THF, 3.0 mmol) in the presence of **1b** (10.4 mg, 2.5 mol%) in Et_2_O (5 mL) at reflux conditions for 72 h (191.5 mg, 80% yield, colorless liquid). ^1^H and ^13^C{^1^H} NMR spectra have been attached in the Supplementary Materials. The product was characterized by comparison with the previously reported ^1^H and ^13^C{^1^H} NMR data [32].

*1-Methoxy-4-[(1E)-3-(4-methylphenyl)prop-1-ene-1-yl]benzene* (**4i**). The compound **4i** was synthesized from 1-methoxy-4-{[(*1E*)-3-(4-methoxyphenoxy)prop-1-en-1-yl]oxy}benzene **2e** (134.3 mg, 0.50 mmol) with **3a** (0.70 mL, 1.1 M in THF, 0.77 mmol) in the presence of **1b** (5.0 mg, 2.5 mol%) in Et_2_O (2.5 mL) at room temperature for 24 h (92.9 mg, 78% yield, colorless liquid). ^1^H and ^13^C{^1^H} NMR spectra have been attached in the Supplementary Materials. The product was characterized by comparison with the previously reported ^1^H and ^13^C{^1^H} NMR data [28].

*1-Fluoro-4-[(1E)-3-(4-methylphenyl)prop-1-ene-1-yl]benzene* (**4j**). The compound **4j** was synthesized from 1-fluoro-4-{[(*1E*)-3-(4-methoxyphenoxy)prop-1-en-1-yl]oxy}benzene **2f** (131.2 mg, 0.51 mmol) with **3a** (1.4 mL, 1.1 M in THF, 1.5 mmol) in the presence of **1b** (5.3 mg, 2.5 mol%) in Et_2_O (2.5 mL) at room temperature for 24 h (102.4 mg, 89% yield, colorless liquid). ^1^H and ^13^C{^1^H} NMR spectra have been attached in the Supplementary Materials. The product was characterized by comparison with the previously reported ^1^H and ^13^C{^1^H} NMR data [33].

*Mixture of 1-methyl-4-[(2E)-5-phenylpent-2-ene-1-yl]benzene* (**4k**) and 1-methyl-4-(5-phenylpent-1 -ene-3-yl)benzene(**5k**). The compounds **4k** and **5k** were synthesized from 1-methoxy-4-{[(*2E*)-5- phenylpent-2-en-1-yl]oxy}benzene **2g** (130.2 mg, 0.49 mmol) with **3a** (0.70 mL, 1.1 M in THF, 0.77 mmol) in the presence of **1b** (5.1 mg, 2.5 mol%) in Et_2_O (2.5 mL) at room temperature for 24 h (110.1 mg, 96% yield, **4k**:**5k** = 54:46, colorless liquid). ^1^H and ^13^C{^1^H} NMR spectra of the mixture of **4k** and **5k** have been attached in the Supplementary Materials. **4k**: ^1^H NMR (500 MHz, CDCl_3_) δ 2.32–2.36 (m, 5H), 2.67–2.71 (m, 2H), 3.22–3.28 (m, 2H), 5.50–5.60 (2H), 7.01–7.03 (m, 2H), 7.07–7.20 (m, 5H), 7.25–7.29 (m, 2H); ^13^C{^1^H} NMR (125 MHz, CDCl_3_) δ 21.0, 34.3, 35.9, 38.6, 125.7, 128.2, 128.3, 128.5, 129.0, 129.8, 130.7, 135.3, 137.8, 140.2, 142.0. The product **5k** was characterized by comparison with the previously reported ^1^H and ^13^C{^1^H} NMR data [34].

The compounds **4k** and **5k** were synthesized from (3-methoxypent-4-en-1-yl)benzene **2i** (174.9 mg, 0.99 mmol) with **3a** (1.4 mL, 1.1 M in THF, 1.5 mmol) in the presence of **1b** (10.1 mg, 2.5 mol%) in Et_2_O (5 mL) at room temperature for 24 h (218.2 mg, 93% yield, **4k**:**5k** = 57:43). 

*1-Methyl-4-[(3E)-4-phenylbut-3-ene-2-yl]benzene* (**4l**). The compound **4l** was synthesized from the mixture of 1-methoxy-4-{[(*3E*)-4-phenylbut-3-en-2-yl]oxy}benzene **2j** and 1-methoxy-4-{[(*2E*)-1-phenylbut-2-en-1-yl]oxy}benzene **2k** (254.7 mg, **2j**:**2k** = 67:33, 1.00 mmol) with **3a** (1.4 mL, 1.1 M in THF, 1.5 mmol) in the presence of **1b** (10.1 mg, 2.5 mol%) in Et_2_O (5 mL) at room temperature for 24 h (200.2 mg, 90% yield, colorless liquid). ^1^H and ^13^C{^1^H} NMR spectra of the mixture of **4l** and **5l** have been attached in the Supplementary Materials. The product **4l** was characterized by comparison with the previously reported ^1^H and ^13^C{^1^H} NMR data [29].

## 4. Conclusions

We have described the cross-coupling reaction of allylic aryl ethers with arylmagnesium reagents catalyzed by nickel(II) pincer complexes **1**. Complex **1b** bearing a β-aminoketonato unit tethering a diphenylphosphino group showed excellent catalyst performance. The reactions of a range of cinnamyl ether derivatives proceeded with high regioselectively to afford solely the linear coupled products in high yield. The coupling reactions of α- and γ-alkyl substituted allylic ethers gave a mixture of linear and branched products. These results indicate that the reaction proceeds via a π-allyl nickel intermediate. Based on stoichiometric reactions, we assumed that an anionic Ni(0) species derived from the reduction of nickel(II) complex **1b** with an excess amount of a Grignard reagent was the active intermediate in the reaction. Further investigations on mechanistic aspects and the coupling reactions of various organometallic reagents with organic electrophiles are currently underway in our laboratory.

## Figures and Tables

**Table 1:** The cross-coupling reaction of **2a** with **3a** using Ni(II) pincer complexes (**1**). ^a^

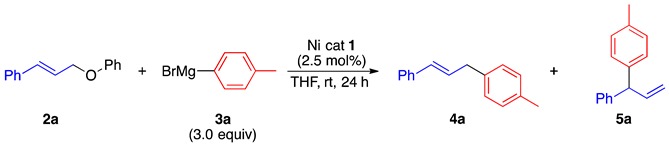				
Ni cat **1** Yield of **4a** (%) ^b^ Recovery of **2a** (%) ^b^	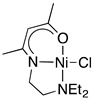 **1a** 7 43	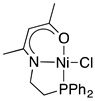 **1b** 86 0	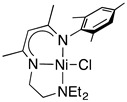 **1c** 2 83	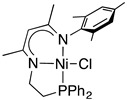 **1d** 4 90

^a^ The reaction was carried out using **2a** (0.5 mmol) and **3a** (1.5 mmol) in the presence of **1** (0.0125 mmol, 2.5 mol%) in THF (5 mL) for 24 h at room temperature. ^b^ The yield of **4a** and recovery of **2a** were determined by ^1^H NMR analysis using pyrazine as the internal standard.

**Table 2:** Solvent screening using the reaction between **2a** and **3a** with complex **1**. ^a^

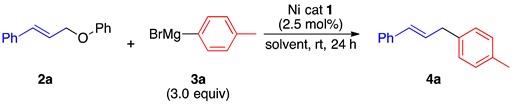							
**Entry**	**Ni Cat 1**	**Solvent**	**4a (%) ^b^**	**Entry**	**Ni Cat 1**	**Solvent**	**4a (%) ^b^**
1	**1b**	THF	86	7	**1b**	DMF	0
2	**1b**	CPME	89	8	**1b**	Et_2_O	99(94) ^c^
3	**1b**	1,4-dioxane	71	9	**1a**	Et_2_O	22
4	**1b**	MTBE	82	10	**1c**	Et_2_O	19
5	**1b**	toluene	83	11	**1d**	Et_2_O	30
6	**1b**	CH_2_Cl_2_	24	12^d^	**1b**	Et_2_O	95(91) ^c^

^a^ The reaction was carried out using **2a** (0.5 mmol) and **3a** (1.5 mmol) in the presence of **1** (0.0125 mmol, 2.5 mol%) in solvent (5 mL) for 24 h at room temperature. ^b^ The yield of **4a** was determined by ^1^H NMR analysis using pyrazine as the internal standard. ^c^ Isolated yield. ^d^ 0.75 mmol of **3a** was used.

**Table 3:** The effect of the leaving group (LG) on the cinnamyl electrophiles. ^a^

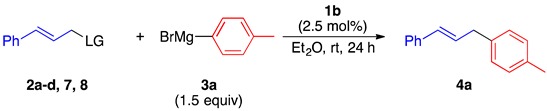		
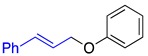	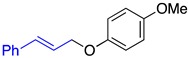	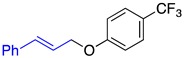
**2a**, 91%, 60% ^b^	**2b**, 96%, 95% ^b^	**2c**, 62% ^c^
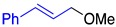	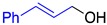	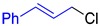
**2d**, 90% ^c^, 73% ^b^	**7**, 40% ^c,d^	**8**, 38% ^c^

^a^ The reaction was carried out using the cinnamyl electrophile (**2**, **7**, **8** (0.5 mmol)), and **3a** (0.75 mmol) in the presence of **1b** (0.0125 mmol, 2.5 mol%) in Et_2_O (5 mL) for 24 h at room temperature. Isolated yield. ^b^ 1 mol% of **1b** was used. ^c^ The yield of **4a** was determined by ^1^H NMR analysis using pyrazine as the internal standard. ^d^ 1.25 mmol of **3a** was used.

**Table 4:** The cross-coupling reaction of allylic ethers **2** with arylmagnesium reagents **3**. ^a^

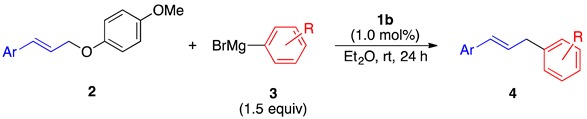		
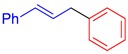	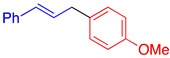	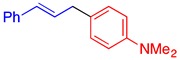
**4b**, 94%	**4c**, 94%	**4d**, 95% ^b^
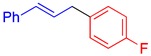	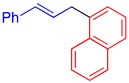	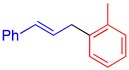
**4e**, 96%	**4f**, 85% ^c^	**4g**, 79% ^d^
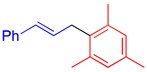	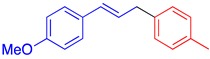	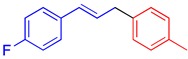
**4h**, 80% ^e^	**4i**, 78% ^b^	**4j**, 89% ^f^

^a^ The reaction was carried out using **2** (0.5 mmol) and **3** (0.75 mmol) in the presence of **1b** (0.005 mmol, 1.0 mol%) in Et_2_O (5 mL) for 24 h at room temperature. Isolated yield. ^b^ 2.5 mol% of **1b** was used. ^c^ 2.5 mol% of **1b** and 1.5 mmol of **3f** were used. The reaction was carried out under reflux conditions for 72 h. ^d^ 2.5 mol% of **1b** and 1.1 mmol of **3g** were used. The reaction was carried out under reflux conditions for 24 h. ^e^ 2.5 mol% of **1b** and 1.5 mmol of **3h** were used. The reaction was carried out under reflux conditions for 72 h. ^f^ 2.5 mol% of **1b** and 1.5 mmol of **3a** were used.

**Table 5:** The cross-coupling reaction of various allylic ethers **2** with **3a**. ^a^

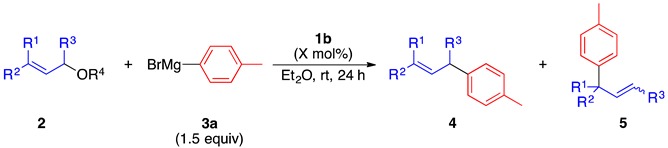					
**Entry**	**2**		**X (mol%)**	**4/5**	**4 + 5 (%)**
1	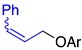	**2b′** (*cis*:*trans* = 86:14)	1.0	**4a**:**5a** = >99:<1	88
2	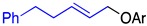	**2g**	2.5	**4k**:**5k** = 54:46	96
3	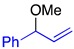	**2h**	2.5	**4a**:**5a** = >99:<1	94
4	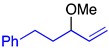	**2i**	2.5	**4k**:**5k** = 57:43	93
5	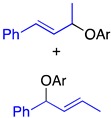	**2j** **2k** (**2j**:**2k** = 67:33)	2.5	**4l**:**5l** = >99:<1	90

^a^ The reaction was carried out using **2** (0.5–1.0 mmol) and **3** (0.75–1.5 mmol) in the presence of **1b** (0.005–0.0125 mmol, 1.0–2.5 mol%) in Et_2_O (5 mL) for 24 h at room temperature. Isolated yield. (Ar = 4-MeOC_6_H_4_).

**Table 6:** The stoichiometric reaction of allylic ether **2b** with aryl Grignard reagent **3a**. ^a^

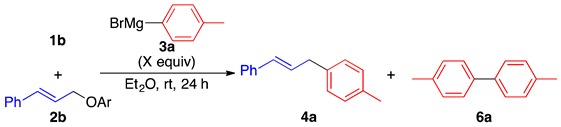				
**Entry**	**X (equiv)**	**4a (%) ^b^**	**6a (mmol) ^c^**	**Recovery of 2b (%) ^b^**
1	1	N.D.	0.03	83
2	2	23	0.09	40
3	3	83	0.19	0
4	4	83	0.23	0

^a^ Reaction conditions: **1b** (0.2 mmol), **2b** (0.2 mmol), and **3a** in Et_2_O (5 mL) at room temperature (Ar = 4-MeOC_6_H_4_). ^b^ The yield of **4a** and recovery of **2b** were determined by ^1^H NMR analysis using pyrazine as the internal standard. ^c^ The amount of **6a** was determined by ^1^H NMR analysis using pyrazine as the internal standard.

## Supplementary Materials

Supplementary materials including ^1^H and ^13^C{^1^H} NMR spectra are available online.

## References

[1] De Meijere A., Diederich F. (2004). Metal-Catalyzed Cross-Coupling Reactions.

[2] Cheng Q., Tu H.-F., Zhen C., Qu J.-P., Helmchen G., You S.-L. (2019). Iridium-Catalyzed Asymmetric Allylic Substitution Reactions. Chem. Rev..

[3] Butt N.A., Zhang X. (2015). Transition metal-catayzed allylic substitution reactions with unactivated allylic substrates. Chem. Soc. Rev..

[4] Sundararaju B., Achard M., Bruneau C. (2012). Transition Metal Catalyzed Nucleophilic Allylic Substitution: activation of allylic alcohols via π-allylic species. Chem. Soc. Rev..

[5] Harutyunyan S.R., den Hartog T., Geurts K., Minnaard A.J., Feringa B.L. (2008). Catalytic Asymmetric Conjugate Addition and Allylic Alkylation with Grignard Reagents. Chem. Rev..

[6] Alexakis A., Bäckvall J.E., Krause N., Pàmies O., Diéguez M. (2008). Enantioselective Copper-Catalyzed Conjugate Addition and Allylic Substitution Reactions. Chem. Rev..

[7] Pérez M., Fañanás-Mastral M., Hornillos V., Rudolph A., Bos P.H., Harutyunyan S.R., Feringa B.L. (2012). Asymmetric Allylic Alkylation of Acyclic Allylic Ethers with Organolithium Reagents. Chem. Eur. J..

[8] Yasui H., Mizutani K., Yorimitsu H., Oshima K. (2006). Cobalt- and rhodium-catalyzed cross-coupling reaction of allylic ethers and halides with organometallic reagents. Tetrahedron.

[9] Gärtner D., Konnerth H., von Wangelin A.J. (2013). Highly practical iron-catalyzed C–O cleavage reactions. Catal. Sci. Technol..

[10] Qi L., Ma E., Jia F., Li Z. (2016). Iron-catalyzed allylic substitution reactions of allylic ethers with Grignard reagents. Tetrahedron Lett..

[11] Ma E., Jiang Y., Chen Y., Qi L., Yan X., Li Z. (2018). Salicylate-Directed C−O Bond Cleavage: Iron-Catalyzed Allylic Substitution with Grignard Reagents. Asian J. Org. Chem..

[12] Seto C., Otsuka T., Takeuchi Y., Tabuchi D., Nagano T. (2018). Iron-Catalyzed Grignard Cross-Couplings with Allylic Methyl Ethers or Allylic Trimethylsilyl Ethers. Synlett.

[13] Tao J.-L., Yang B., Wang Z.-X. (2015). Pincer-Nickel-Catalyzed Allyl-Aryl Coupling between Allyl Methyl Ethers and Arylzinc Chlorides. J. Org. Chem..

[14] Gossage R.A., van de Kuil L., van Koten G. (1998). Diaminoarylnickel(II) “Pincer” Complexes: Mechanistic Considerations in the Kharasch Addition Reaction, Controlled Polymerization, and Dendrimeric Transition Metal Catalysts. Acc. Chem. Res..

[15] Albrecht M., van Koten G. (2001). Platinum Group Organometallics Based on “Pincer” Complexes: Sensors, Switches, and Catalysts. Angew. Chem. Int. Ed..

[16] Van der Boom M.E., Milstein D. (2003). Cyclometalated Phosphine-Based Pincer Complexes: Mechanistic Insight in Catalysis, Coordination, and Bond Activation. Chem. Rev..

[17] Nishiyama H. (2007). Synthesis and use of bisoxazolinyl-phenyl pincers. Chem. Soc. Rev..

[18] Benito-Garagorri D., Kirchner K. (2008). Modularly Designed Transition Metal PNP and PCP Pincer Complexes based on Aminophosphines: Synthesis and Catalytic Applications. Acc. Chem. Res..

[19] Wang Z.-X., Liu N. (2012). Nickel-Catalyzed Cross-Coupling with Pincer Ligands. Eur. J. Inorg. Chem..

[20] Choi J., MacArthur A.H.R., Brookhart M., Goldman A.S. (2011). Dehydrogenation and Related Reactions Catalyzed by Iridium Pincer Complexes. Chem. Rec..

[21] Selander N., Szabó K. (2011). Catalysis by Palladium Pincer Complexes. Chem. Rec..

[22] Niu J.-L., Hao X.-Q., Gong J.-F., Song M.-P. (2011). Symmetrical and unsymmetrical pincer complexes with group 10 metals: synthesis via aryl C–H activation and some catalytic applications. Dalton Trans..

[23] Schneider S., Meiners J., Askevold B. (2012). Cooperative Aliphatic PNP Amido Pincer Ligands – Versatile Building Blocks for Coordination Chemistry and Catalysis. Eur. J. Inorg. Chem..

[24] Asay M., Morales-Morales D. (2015). Non-symmetric pincer ligands: complexes and applications in catalysis. Dalton Trans..

[25] Murugesan S., Kirchner K. (2016). Non-precious metal complexes with an anionic PCP pincer architecture. Dalton Trans..

[26] Asano E., Hatayama Y., Kurisu N., Ohtani A., Hashimoto T., Kurihara Y., Ueda K., Ishihara S., Nagano H., Yamaguchi Y. (2018). Acetylacetonato-based pincer-type nickel(II) complexes: Synthesis and catalysis in cross-couplings of aryl chlorides with aryl Grignard reagents. Dalton Trans..

[27] Kurisu N., Asano E., Hatayama Y., Kurihara Y., Hashimoto T., Funatsu K., Ueda K., Yamaguchi Y. (2018). A β-diketiminato-based pincer-type nickel(II) complex: Synthesis and catalytic performance in the cross-coupling of aryl fluorides with aryl Grignard reagents. Eur. J. Inorg. Chem..

[28] Yang B., Wang Z.-X. (2017). Nickel-Catalyzed Cross-Coupling of Allyl Alcohols with Aryl- or Alkenylzinc Reagents. J. Org. Chem..

[29] Jia X.-G., Guo P., Duan J., Shu X.-Z. (2018). Dual nickel and Lewis acid catalysis for cross-electrophile coupling: the allylation of aryl halides with allylic alcohols. Chem. Sci..

[30] Cao H., Jiang H., Feng H., Kwan J.M.C., Liu X., Wu J. (2018). Photo-induced Decarboxylative Heck-Type Coupling of Unactivated Aliphatic Acids and Terminal Alkenes in the Absence of Sacrificial Hydrogen Acceptors. J. Am. Chem. Soc..

[31] Nishikata T., Lipshutz B.H. (2009). Allylic Ethers as Educts for Suzuki-Miyaura Couplings in Water at Room Temperature. J. Am. Chem. Soc..

[32] Mino T., Kogure T., Abe T., Koizumi T., Fujita T., Sakamoto M. (2013). Palladium-Catalyzed Allylic Arylation of Allylic Ethers with Arylboronic Acids Using Hydrazone Ligands. Eur. J. Org. Chem..

[33] Zhang Y., Yin S.-C., Lu J.-M. (2015). *N*-Heterocyclic carbene-palladium(II)-1-methylimidazole complex catalyzed allyl-aryl coupling of allylic alcohols with arylboronic acids in neat water. Tetrahedron.

[34] Evans P.A., Uraguchi D. (2003). Regio- and Enantiospecific Rhodium-Catalyzed Arylation of Unsymmetrical Fluorinated Acyclic Allylic Carbonates: Inversion of Absolute Configuration. J. Am. Chem. Soc..

