# Supplementary material for: Cross-Coupling Reaction of Allylic Ethers with Aryl Grignard Reagents Catalyzed by a Nickel Pincer Complex
Source: Molecules. 2019 Jun 21;24(12):2296. doi: 10.3390/molecules24122296 (PMC6631569; doi:10.3390/molecules24122296)

Supporting Information For:

Cross-coupling Reaction of Allylic Ethers with Aryl Grignard Reagents Catalysed by a Nickel Pincer Complex

Toru Hashimoto,* Kei Funatsu, Atsufumi Ohtani, Erika Asano, and Yoshitaka Yamaguchi*

Department of Advanced Materials Chemistry, Graduate School of Engineering, Yokohama National University, 79-5 Tokiwadai, Hodogaya-ku, Yokohama 240-8501, Japan

Contents

^{1}H NMR and ^{13}C{^{1}H} NMR spectra of coupling product **4a** S-2

^{1}H NMR and ^{13}C{^{1}H} NMR spectra of coupling product **4b** S-3

^{1}H NMR and ^{13}C{^{1}H} NMR spectra of coupling product **4c** S-4

^{1}H NMR and ^{13}C{^{1}H} NMR spectra of coupling product **4d** S-5

^{1}H NMR and ^{13}C{^{1}H} NMR spectra of coupling product **4e** S-6

^{1}H NMR and ^{13}C{^{1}H} NMR spectra of coupling product **4f** S-7

^{1}H NMR and ^{13}C{^{1}H} NMR spectra of coupling product **4g** S-8

^{1}H NMR and ^{13}C{^{1}H} NMR spectra of coupling product **4h** S-9

^{1}H NMR and ^{13}C{^{1}H} NMR spectra of coupling product **4i** S-10

^{1}H NMR and ^{13}C{^{1}H} NMR spectra of coupling product **4j** S-11

^{1}H NMR and ^{13}C{^{1}H} NMR spectra of coupling products **4k** and **5k** S-12

^{1}H NMR and ^{13}C{^{1}H} NMR spectra of coupling product **4l** S-13

^{1}H NMR and ^{13}C NMR spectra of coupling products

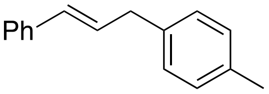

(4a)

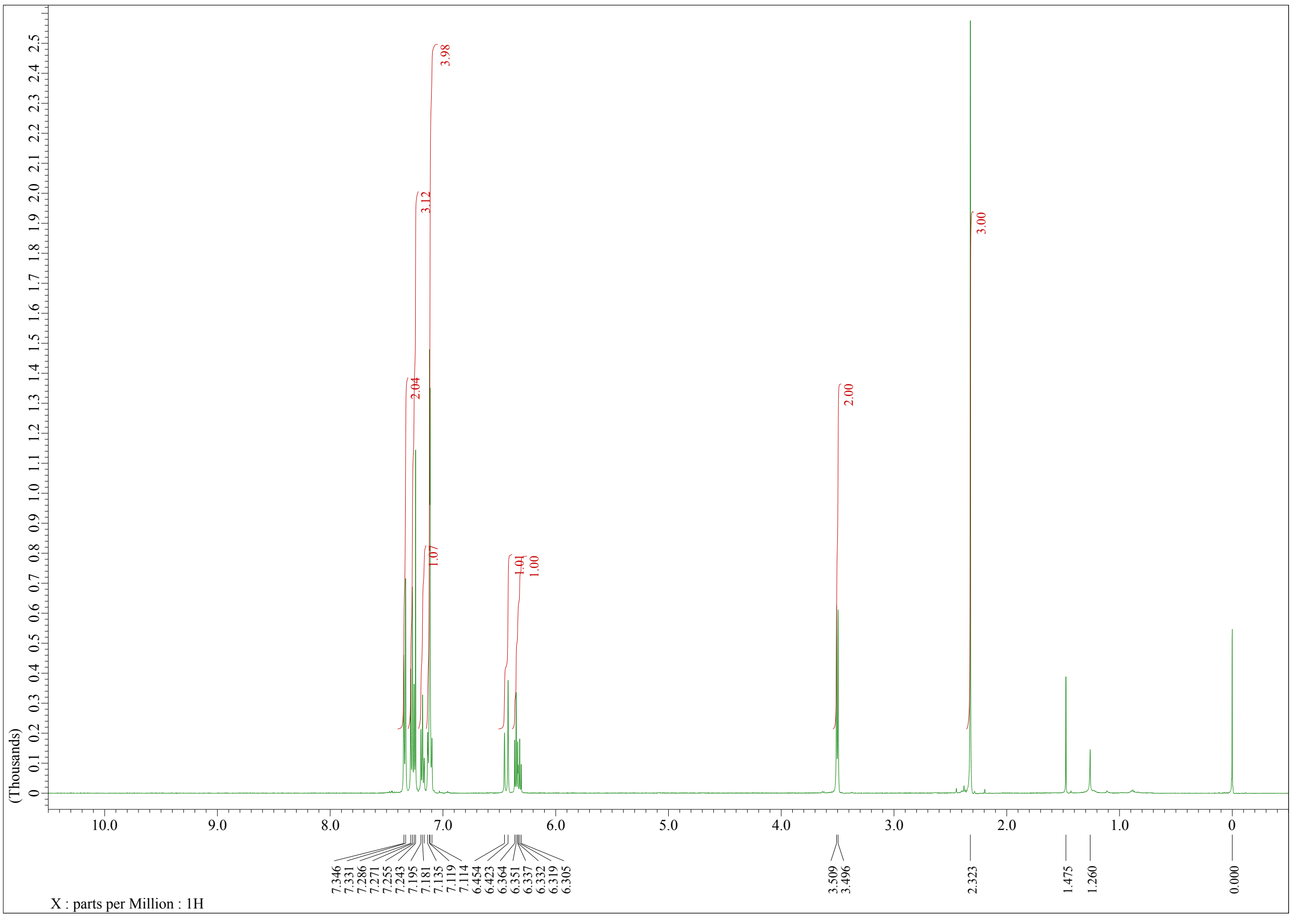

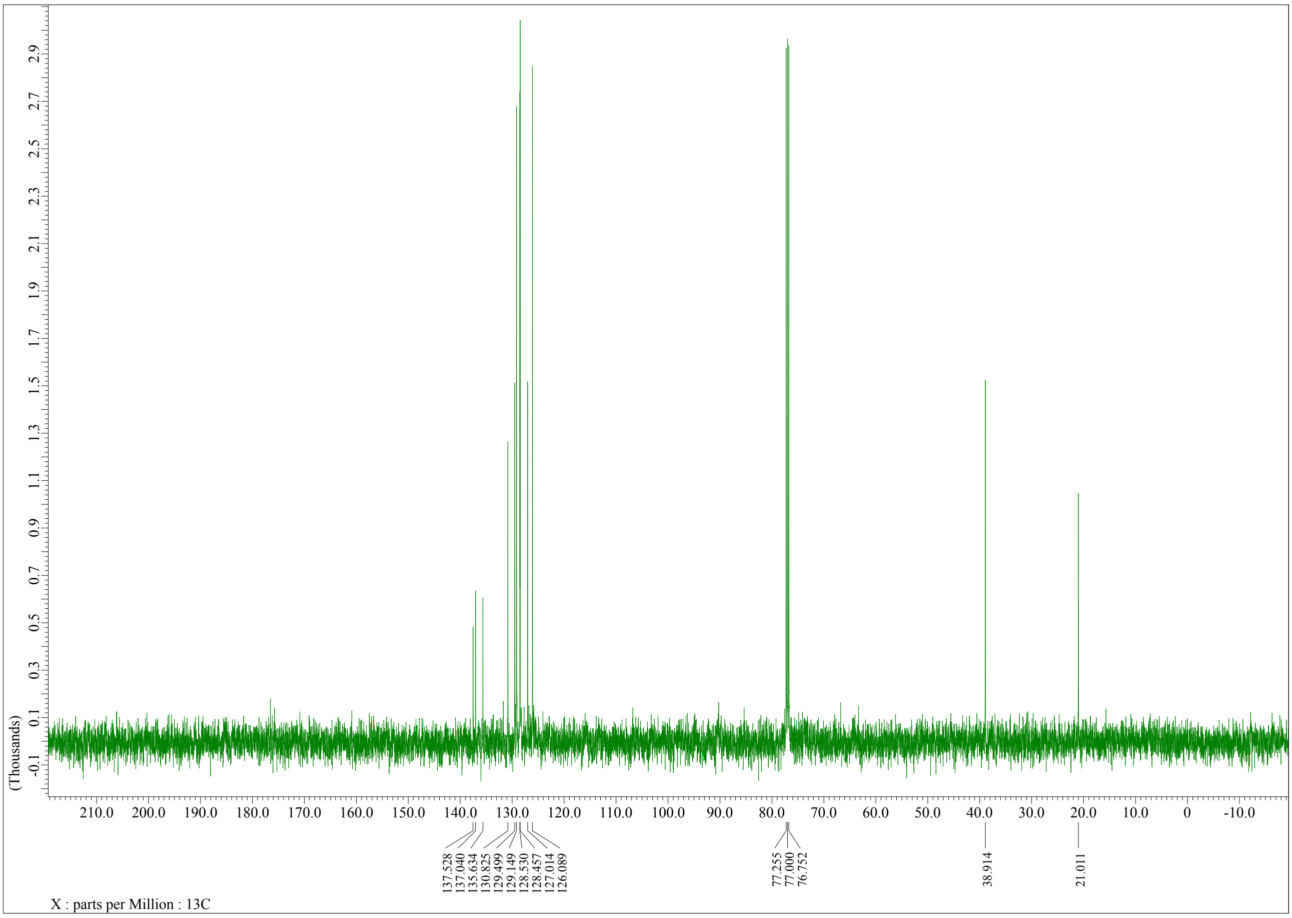

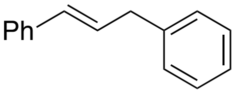
Ph
(**4b**)

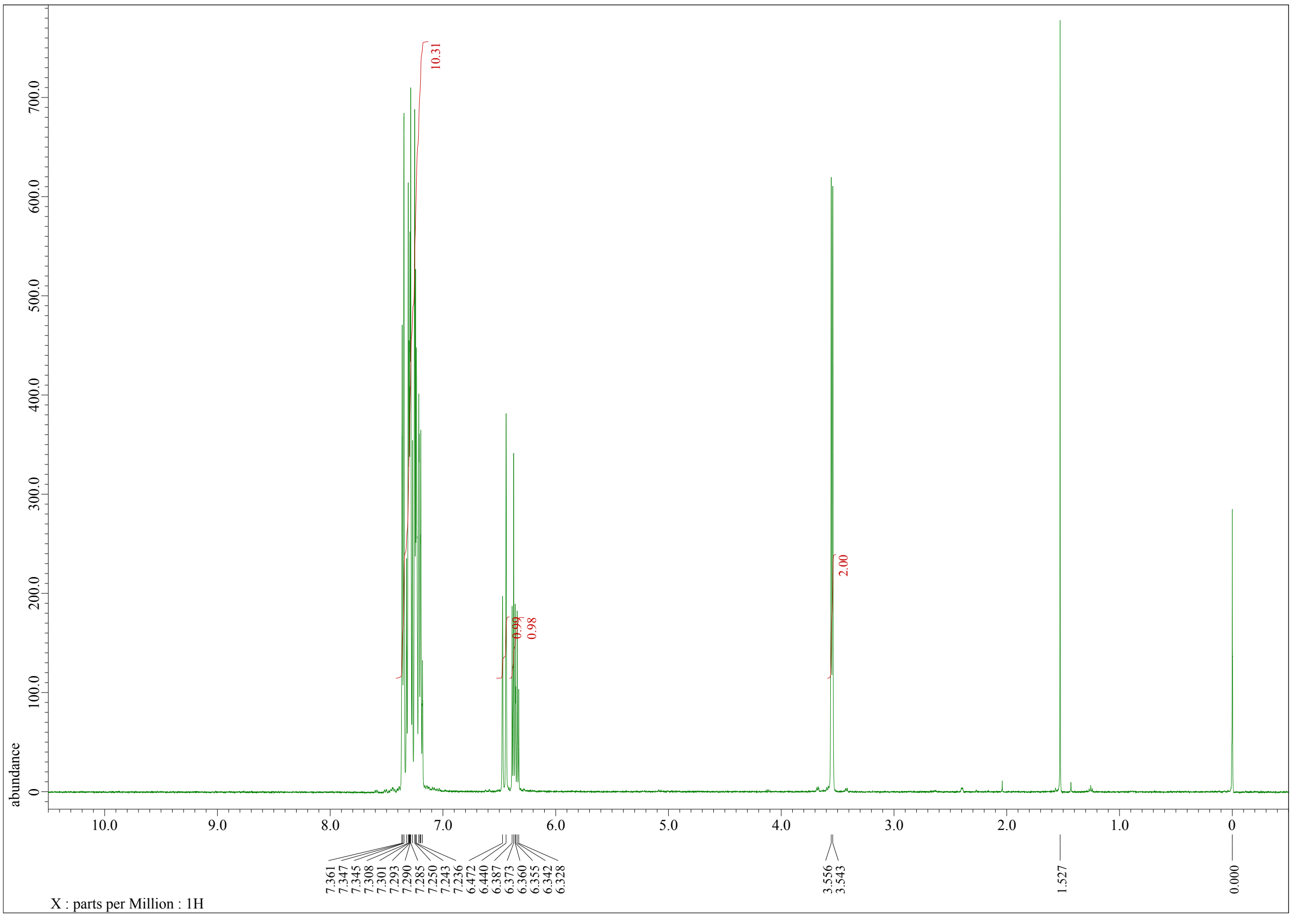
abundance
0
100.0
200.0
300.0
400.0
500.0
600.0
700.0
10.31
0.99
0.98
2.00
10.0
9.0
8.0
7.0
6.0
5.0
4.0
3.0
2.0
1.0
0
7.361
7.347
7.345
7.308
7.301
7.293
7.290
7.285
7.250
7.243
7.236
6.472
6.440
6.387
6.373
6.360
6.355
6.342
6.328
3.556
3.543
1.527
0.000
X : parts per Million : 1H

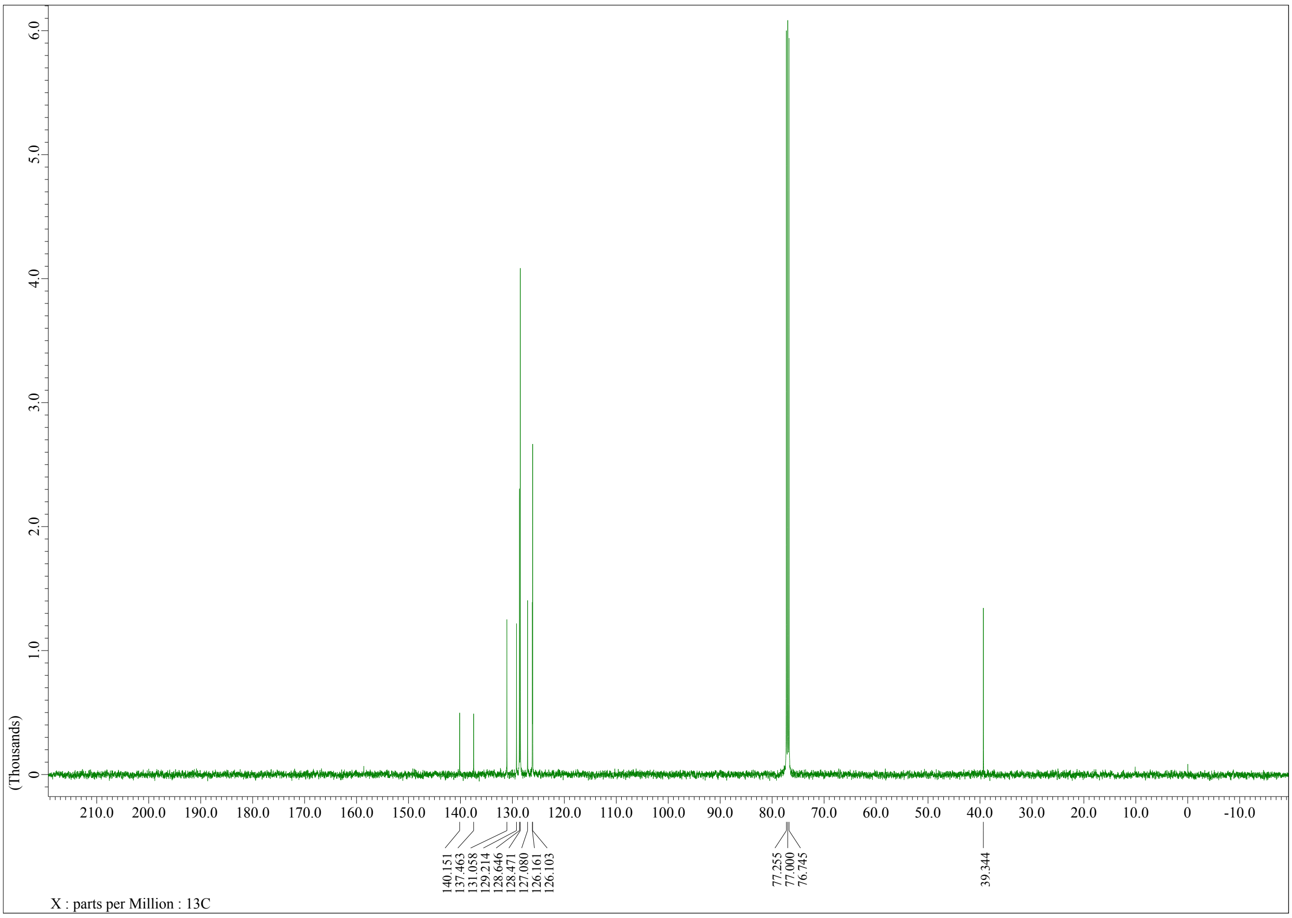
(Thousands)
0
1.0
2.0
3.0
4.0
5.0
6.0
210.0
200.0
190.0
180.0
170.0
160.0
150.0
140.0
130.0
120.0
110.0
100.0
90.0
80.0
70.0
60.0
50.0
40.0
30.0
20.0
10.0
0
-10.0
140.151
137.463
131.058
129.214
128.646
128.471
127.080
126.161
126.103
77.255
77.000
76.745
39.344
X : parts per Million : 13C

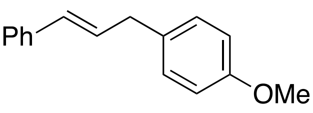

Ph
OMe

(**4c**)

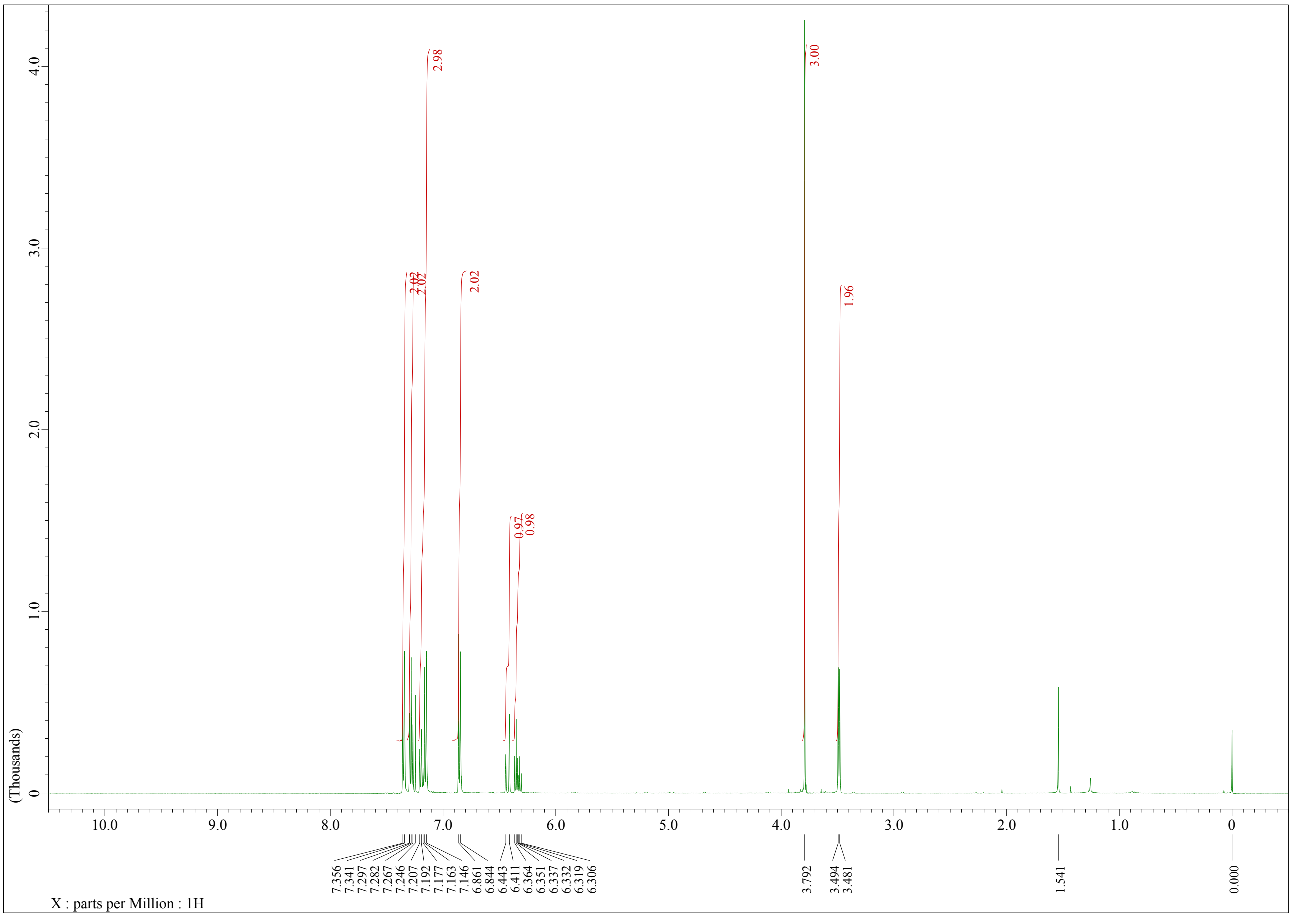

X : parts per Million : 1H
(Thousands)
7.356
7.341
7.297
7.282
7.267
7.246
7.207
7.192
7.177
7.163
7.146
6.861
6.844
6.443
6.411
6.364
6.351
6.337
6.332
6.319
6.306
3.792
3.494
3.481
1.541
0.000

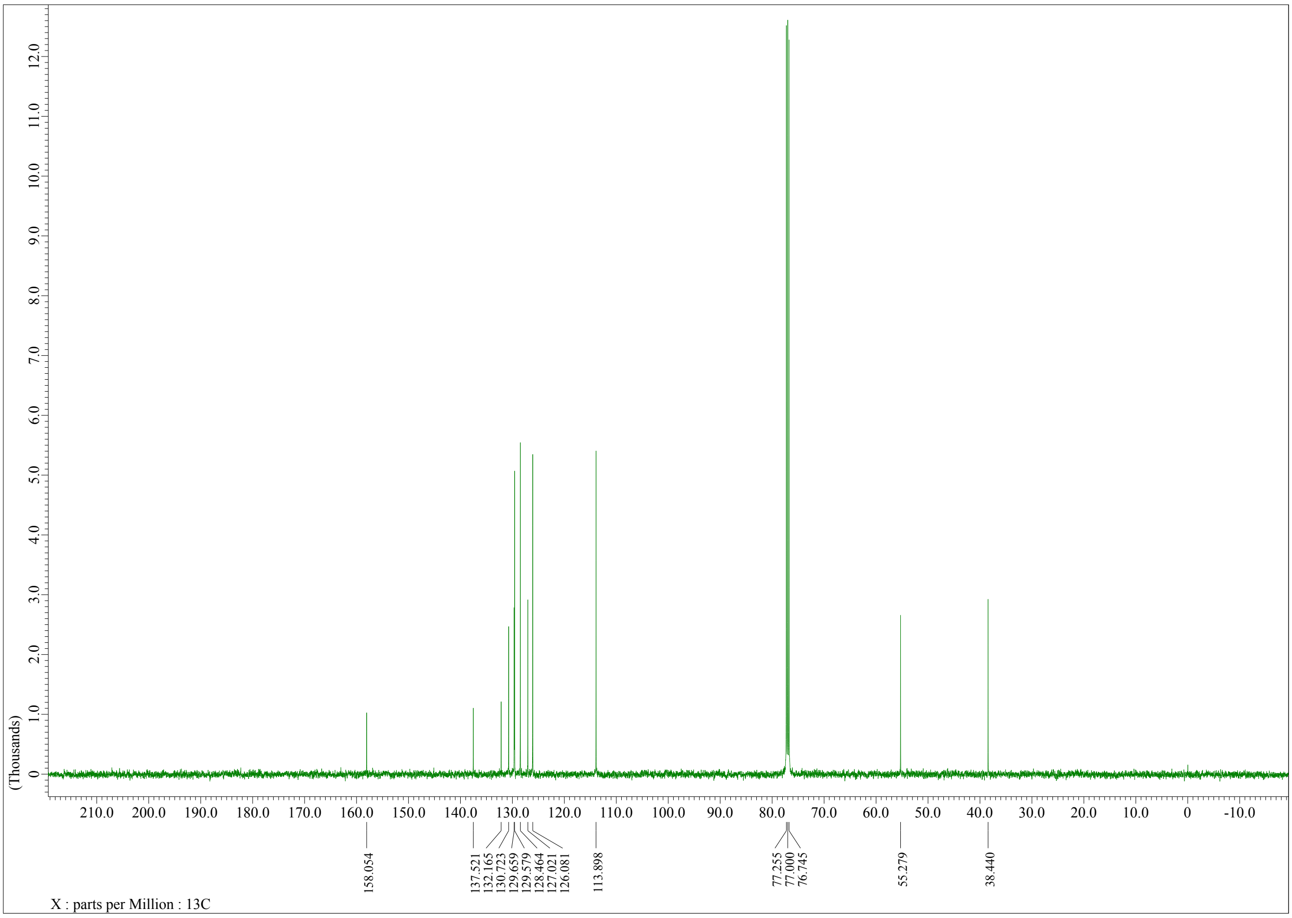

X : parts per Million : 13C
(Thousands)
158.054
137.521
132.165
130.723
129.659
129.579
128.464
127.021
126.081
113.898
77.255
77.000
76.745
55.279
38.440

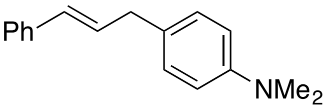
Ph
NMe2
(**4d**)

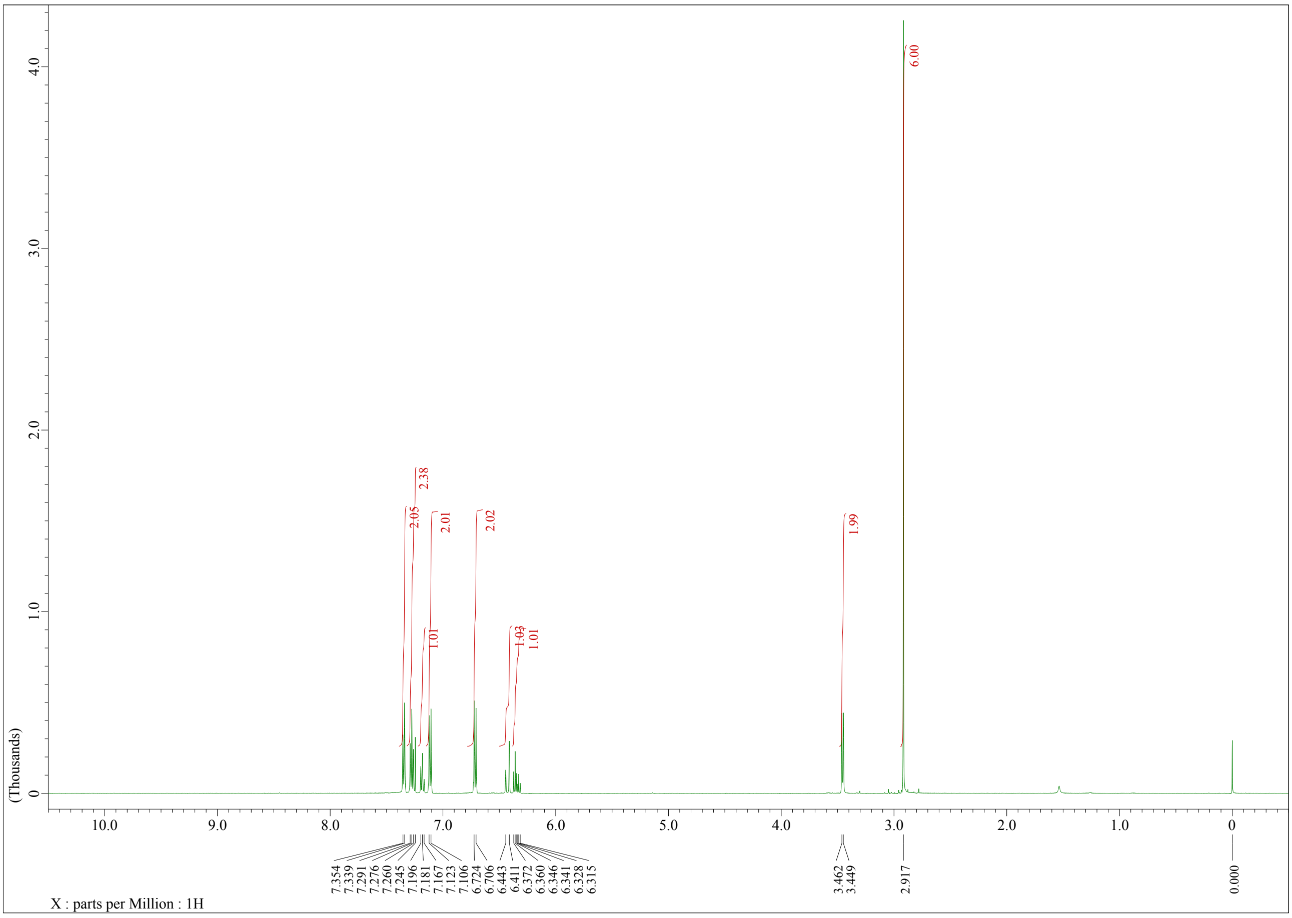
(Thousands)
4.0
3.0
2.0
1.0
0
2.38
2.05
2.01
2.02
1.01
1.03
1.01
1.99
6.00
10.0
9.0
8.0
7.0
6.0
5.0
4.0
3.0
2.0
1.0
0
7.354
7.339
7.291
7.276
7.260
7.245
7.196
7.181
7.167
7.123
7.106
6.724
6.706
6.443
6.411
6.372
6.360
6.346
6.341
6.328
6.315
3.462
3.449
2.917
0.000
X : parts per Million : 1H

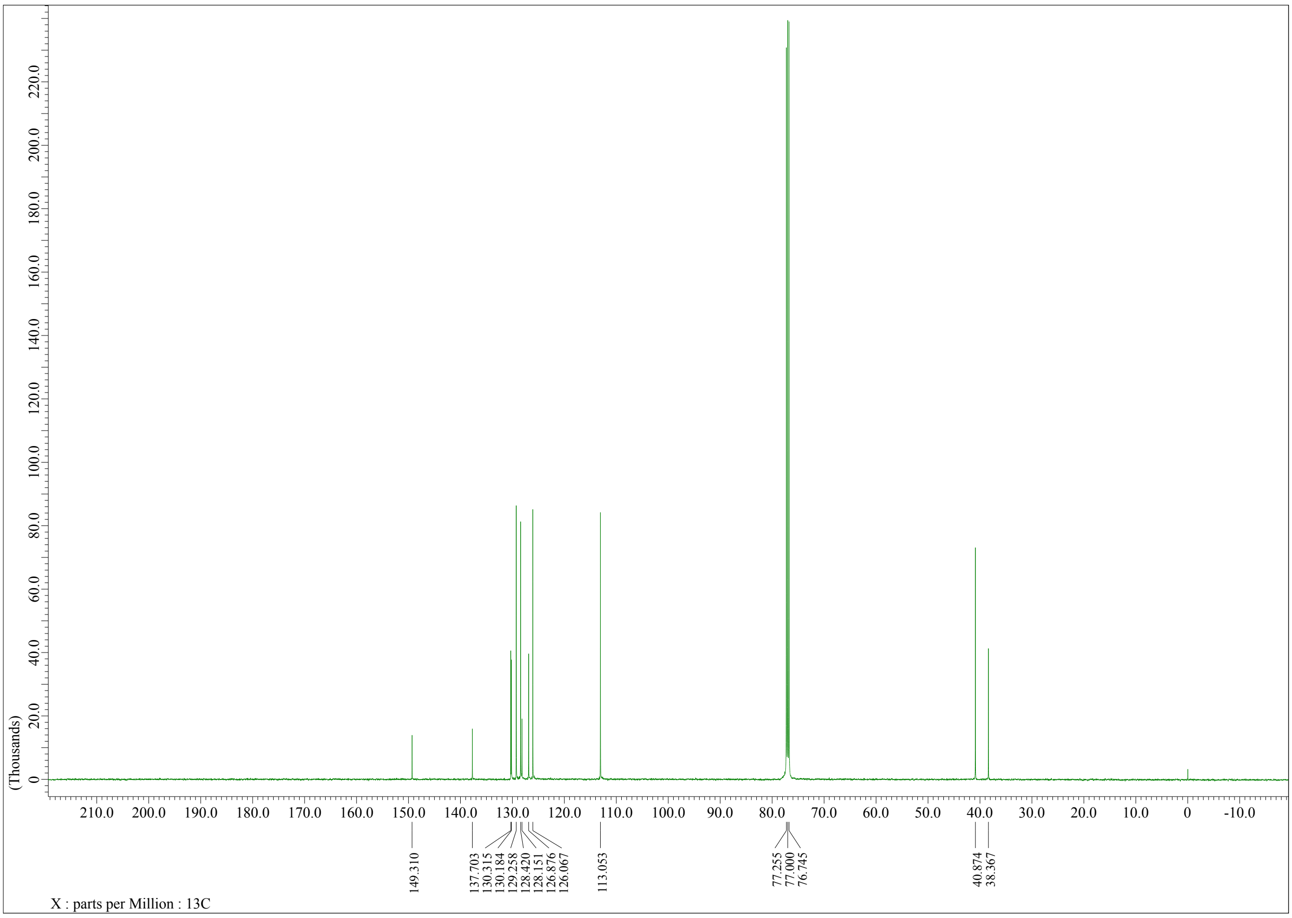
(Thousands)
220.0
200.0
180.0
160.0
140.0
120.0
100.0
80.0
60.0
40.0
20.0
0
210.0
200.0
190.0
180.0
170.0
160.0
150.0
140.0
130.0
120.0
110.0
100.0
90.0
80.0
70.0
60.0
50.0
40.0
30.0
20.0
10.0
0
-10.0
149.310
137.703
130.315
130.184
129.258
128.420
128.151
126.876
126.067
113.053
77.255
77.000
76.745
40.874
38.367
X : parts per Million : 13C

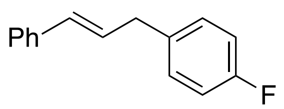
Ph
F

(4e)

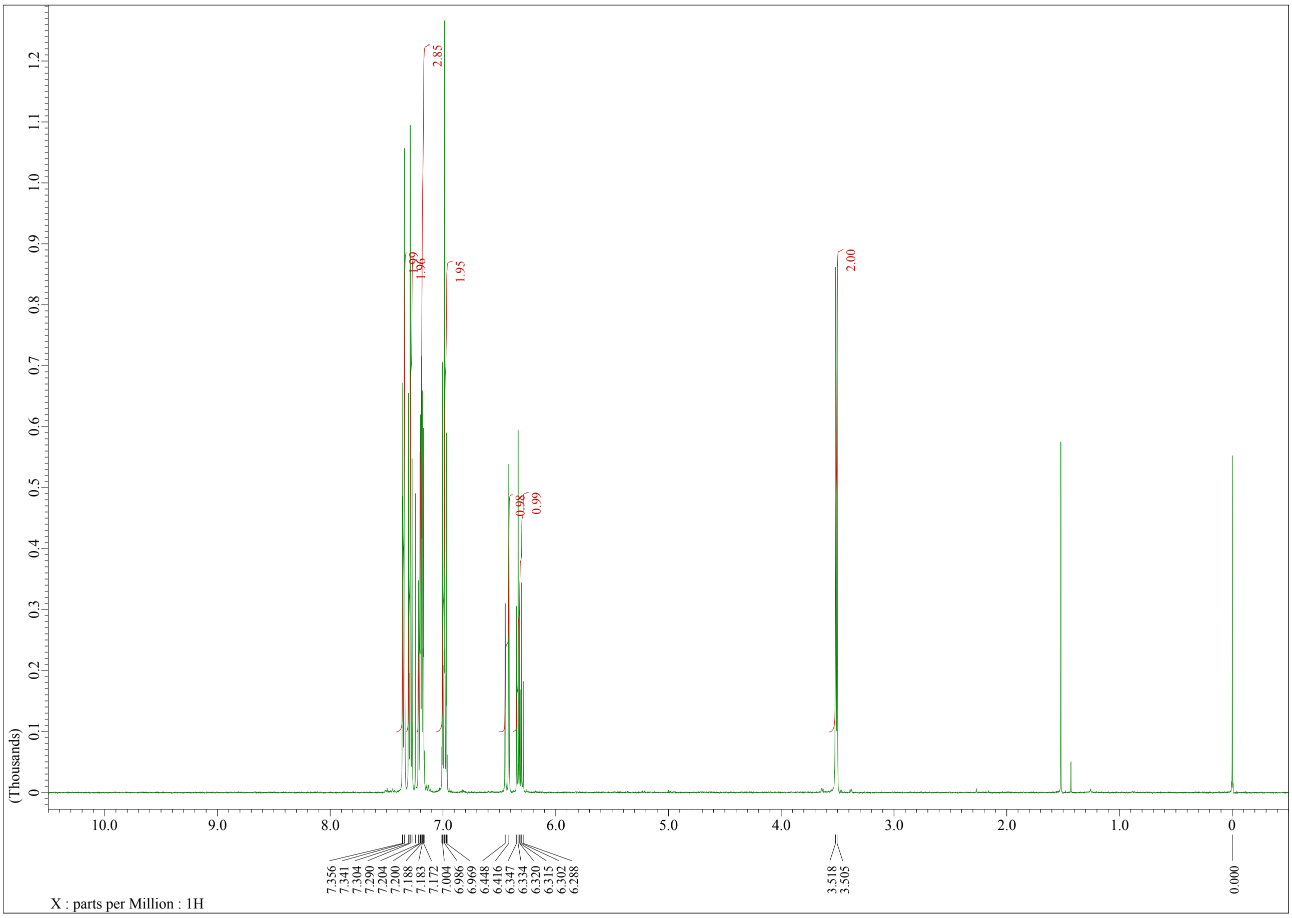
X : parts per Million : 1H
(Thousands)
7.356
7.341
7.304
7.290
7.204
7.200
7.188
7.183
7.172
7.004
6.986
6.969
6.448
6.416
6.347
6.334
6.320
6.315
6.302
6.288
3.518
3.505
0.000
2.85
1.99
1.96
1.95
0.98
0.99
2.00

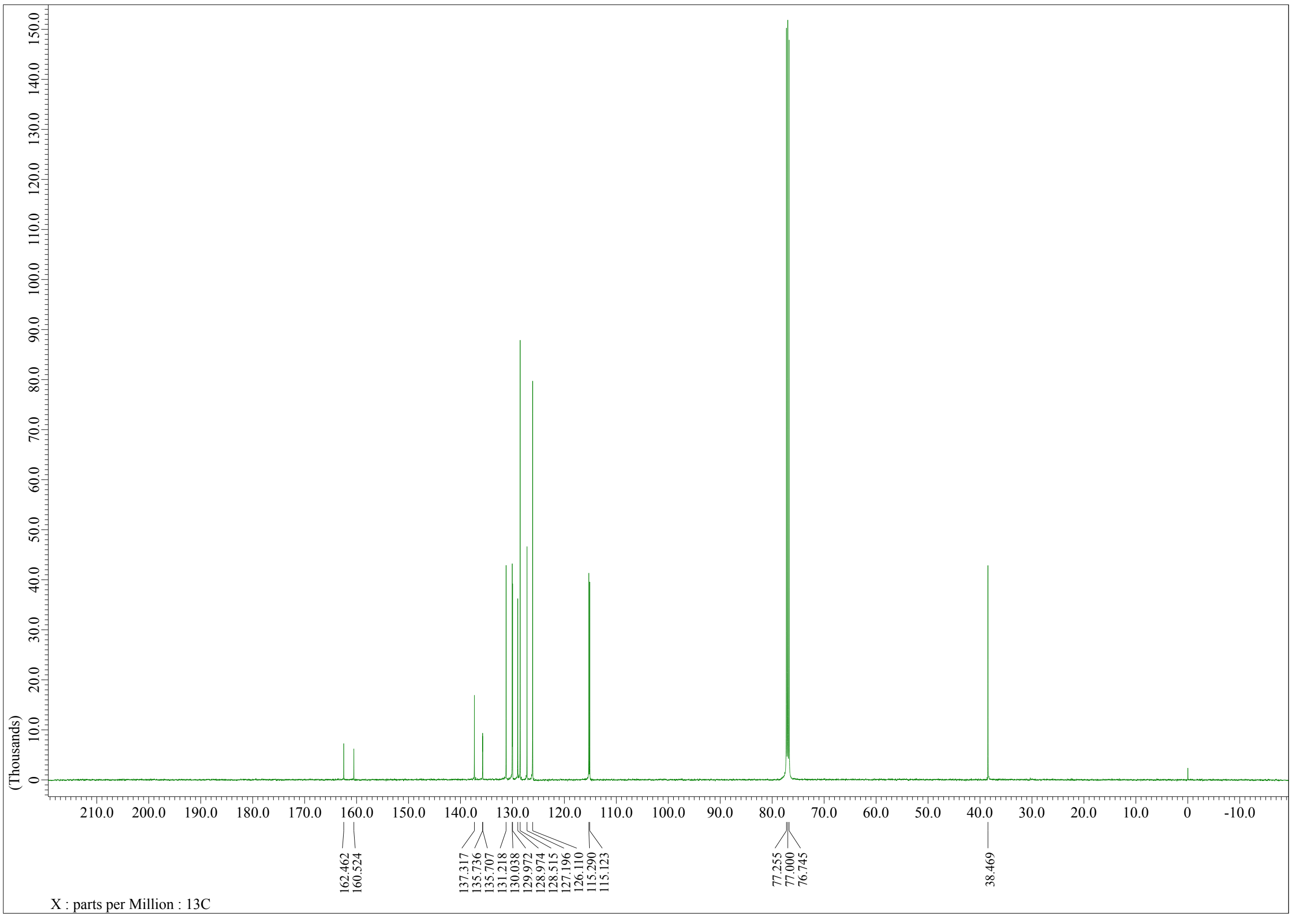
X : parts per Million : 13C
(Thousands)
162.462
160.524
137.317
135.736
135.707
131.218
130.038
129.972
128.974
128.515
127.196
126.110
115.290
115.123
77.255
77.000
76.745
38.469

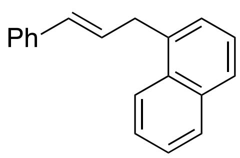
Ph

(4f)

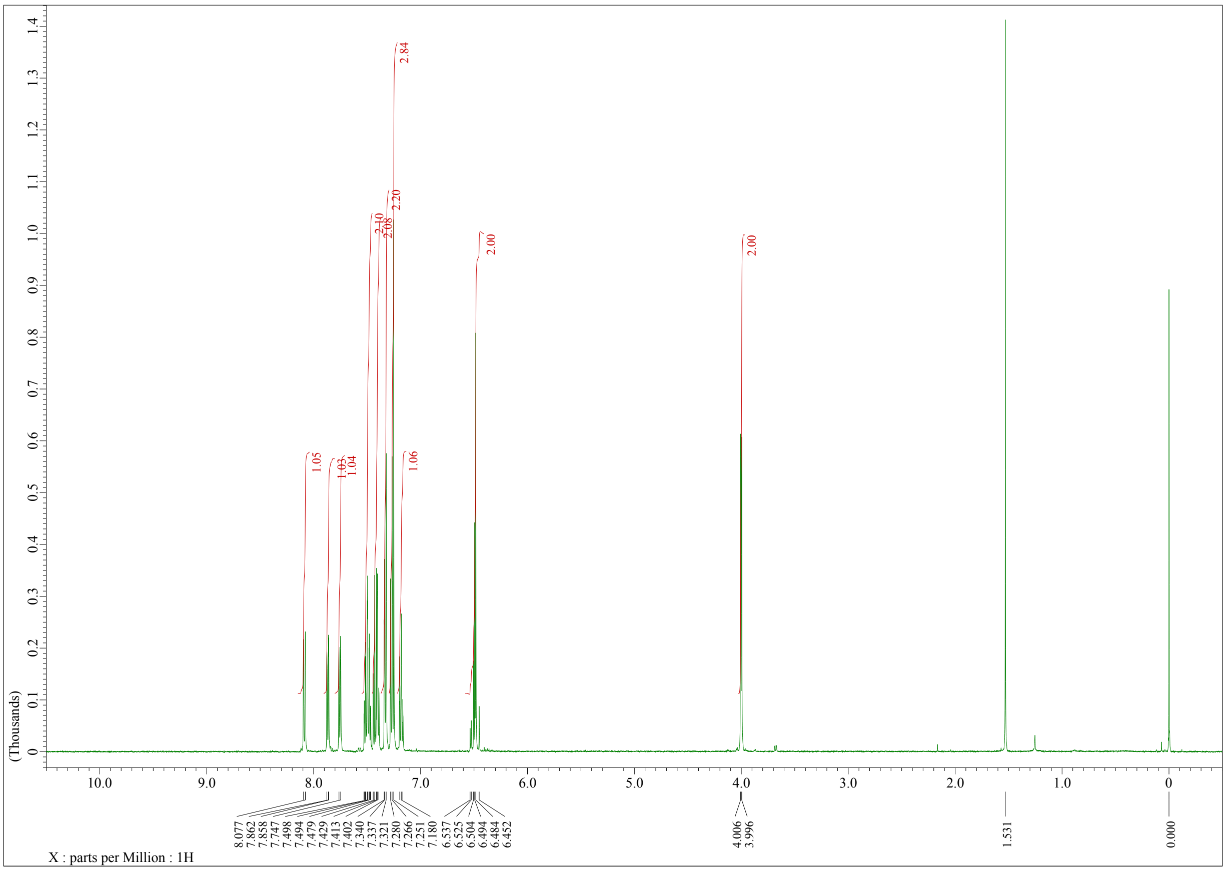
(Thousands)
X : parts per Million : 1H
8.077
7.862
7.858
7.747
7.498
7.494
7.479
7.429
7.413
7.402
7.340
7.337
7.321
7.280
7.266
7.251
7.180
6.537
6.525
6.504
6.494
6.484
6.452
4.006
3.996
1.531
0.000
1.05
1.03
1.04
2.10
2.08
2.20
2.84
1.06
2.00
2.00

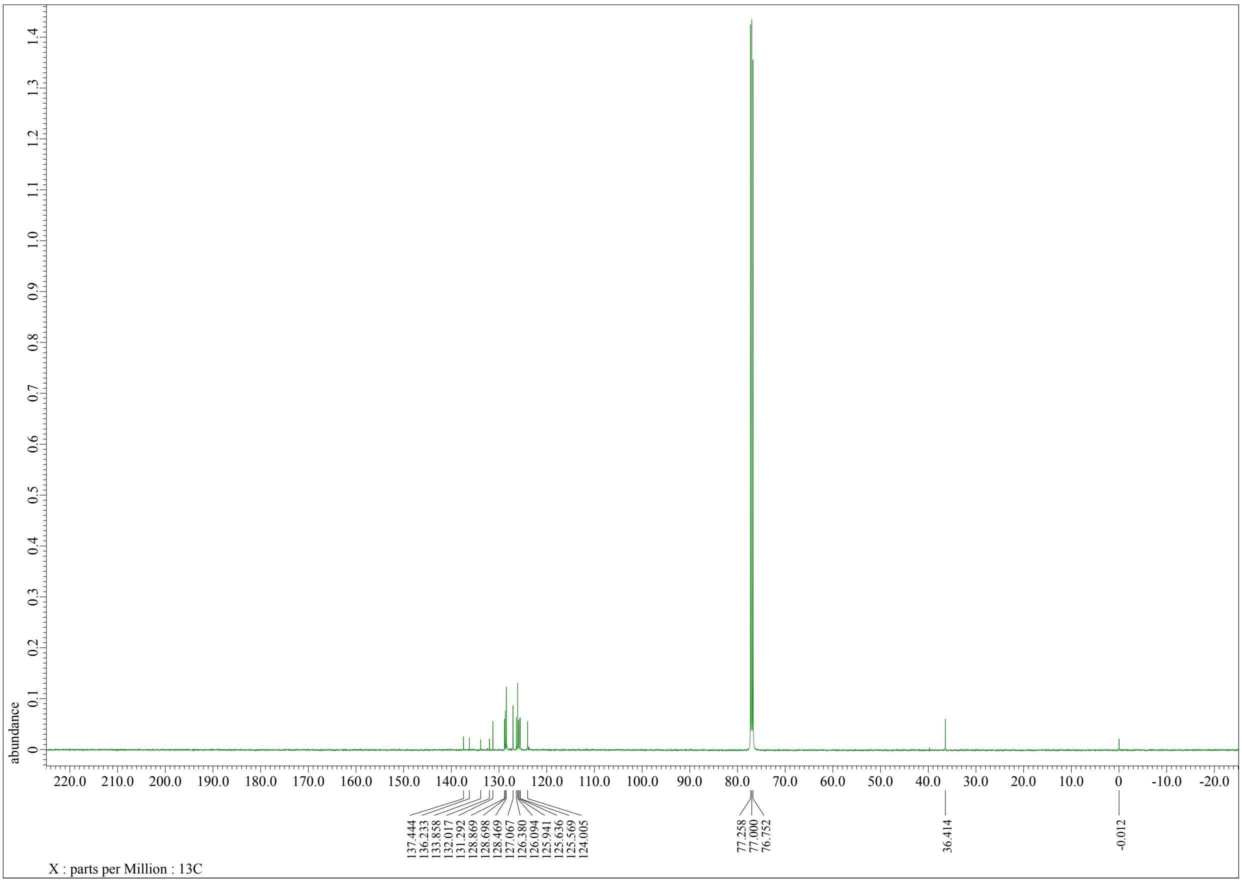
abundance
X : parts per Million : 13C
137.444
136.233
133.858
132.017
131.292
128.869
128.698
128.469
127.067
126.380
126.094
125.941
125.636
125.569
124.005
77.258
77.000
76.752
36.414
-0.012

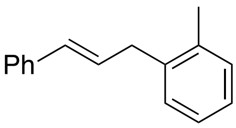
Ph

(4g)

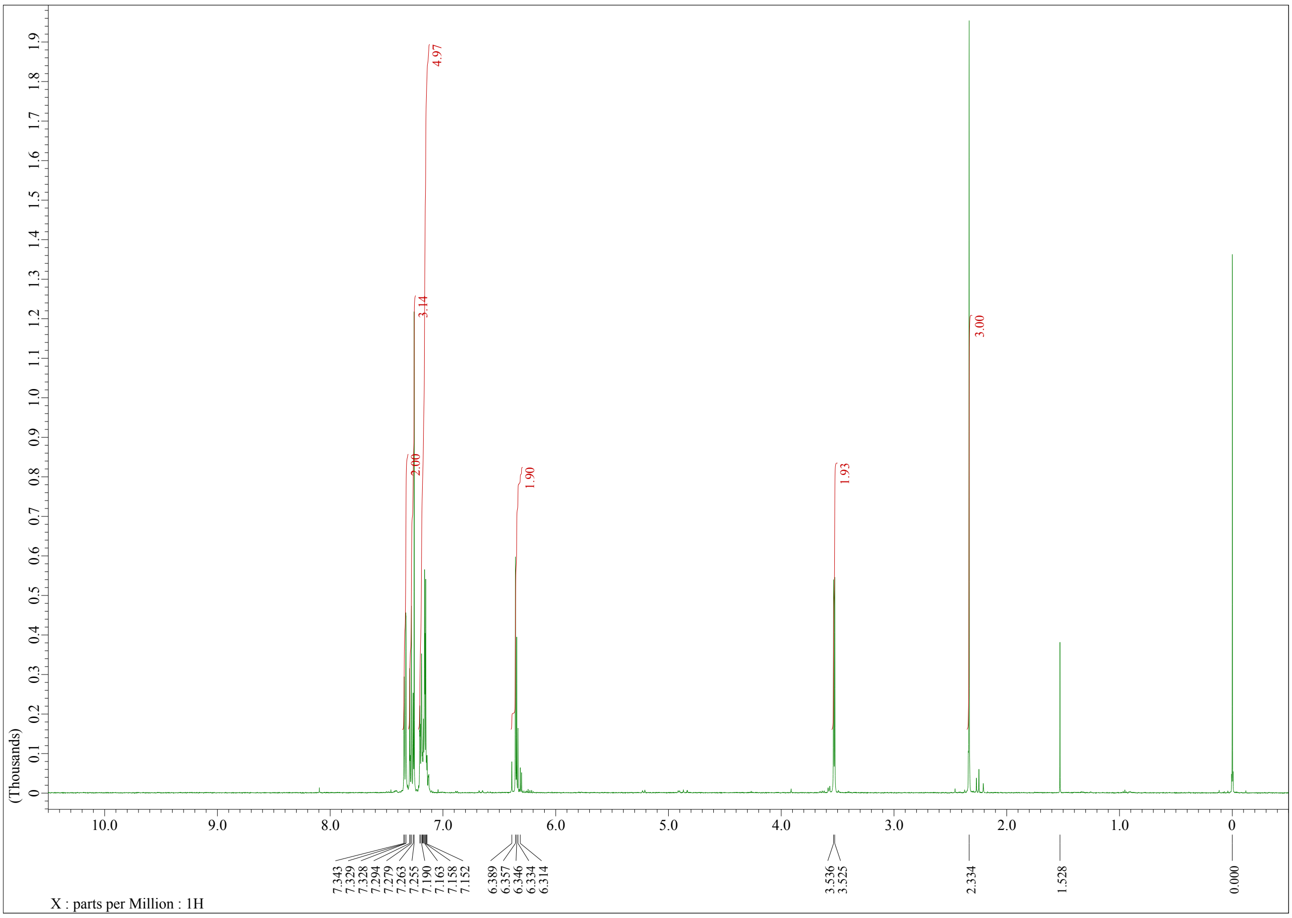
(Thousands)
4.97
3.14
2.00
1.90
1.93
3.00
7.343
7.329
7.328
7.294
7.279
7.263
7.255
7.190
7.163
7.158
7.152
6.389
6.357
6.346
6.334
6.314
3.536
3.525
2.334
1.528
0.000
X : parts per Million : 1H

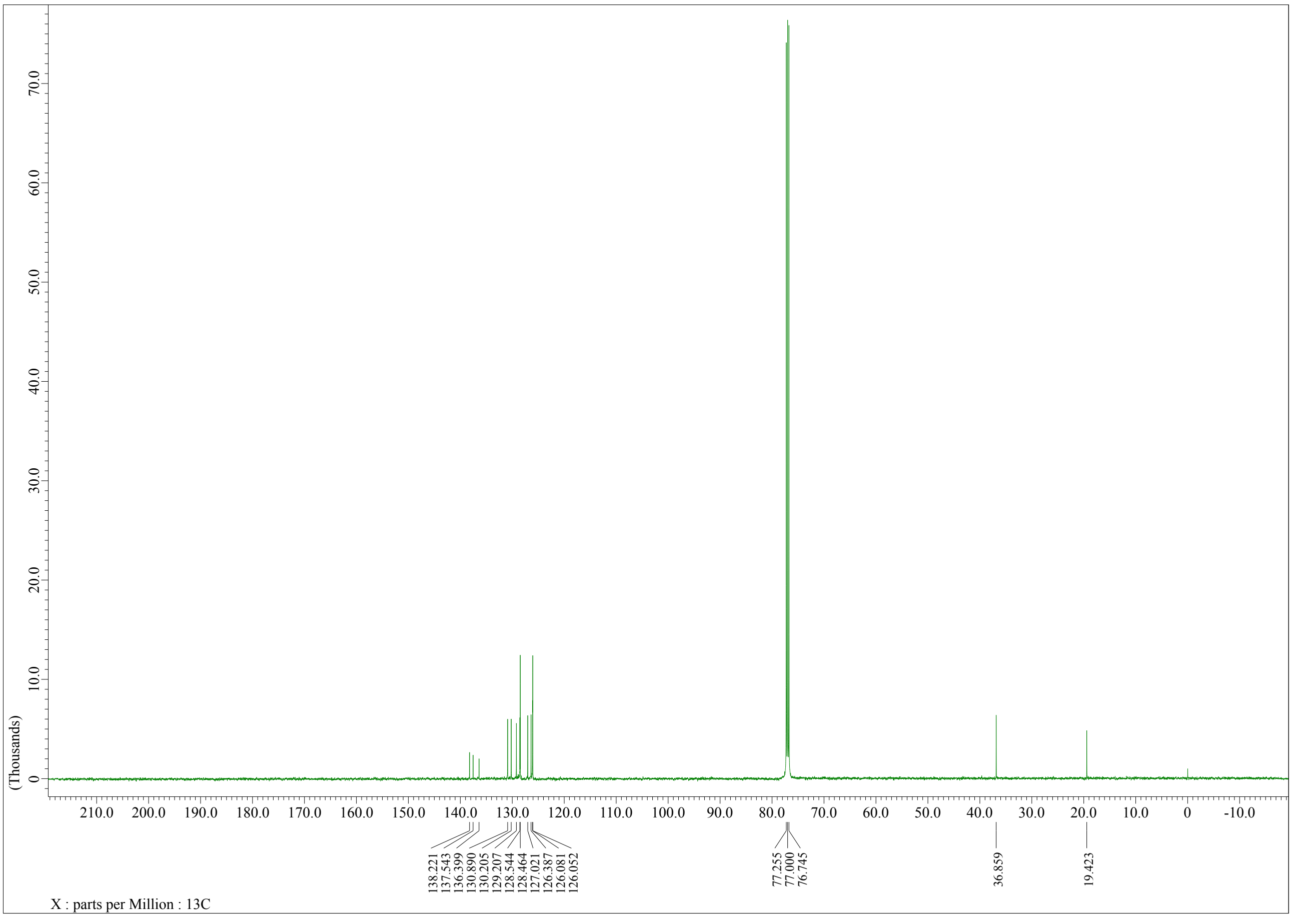
(Thousands)
138.221
137.543
136.399
130.890
130.205
129.207
128.544
128.464
127.021
126.387
126.081
126.052
77.255
77.000
76.745
36.859
19.423
X : parts per Million : 13C

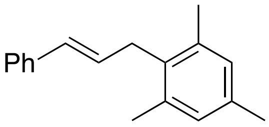
Ph

(**4h**)

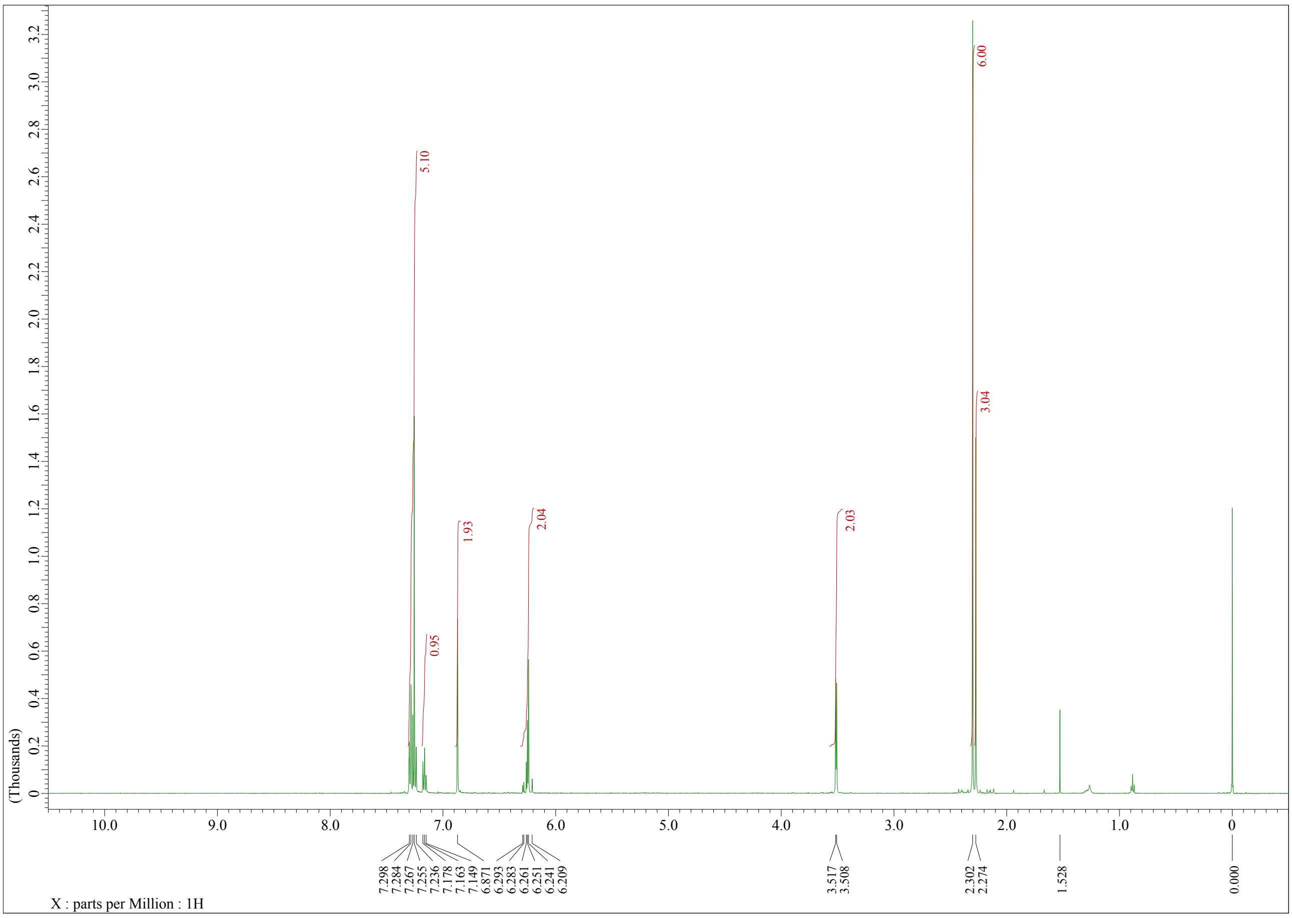
5.10
0.95
1.93
2.04
2.03
6.00
3.04
(Thousands)
7.298
7.284
7.267
7.255
7.236
7.178
7.163
7.149
6.871
6.293
6.283
6.261
6.251
6.241
6.209
3.517
3.508
2.302
2.274
1.528
0.000
X : parts per Million : 1H

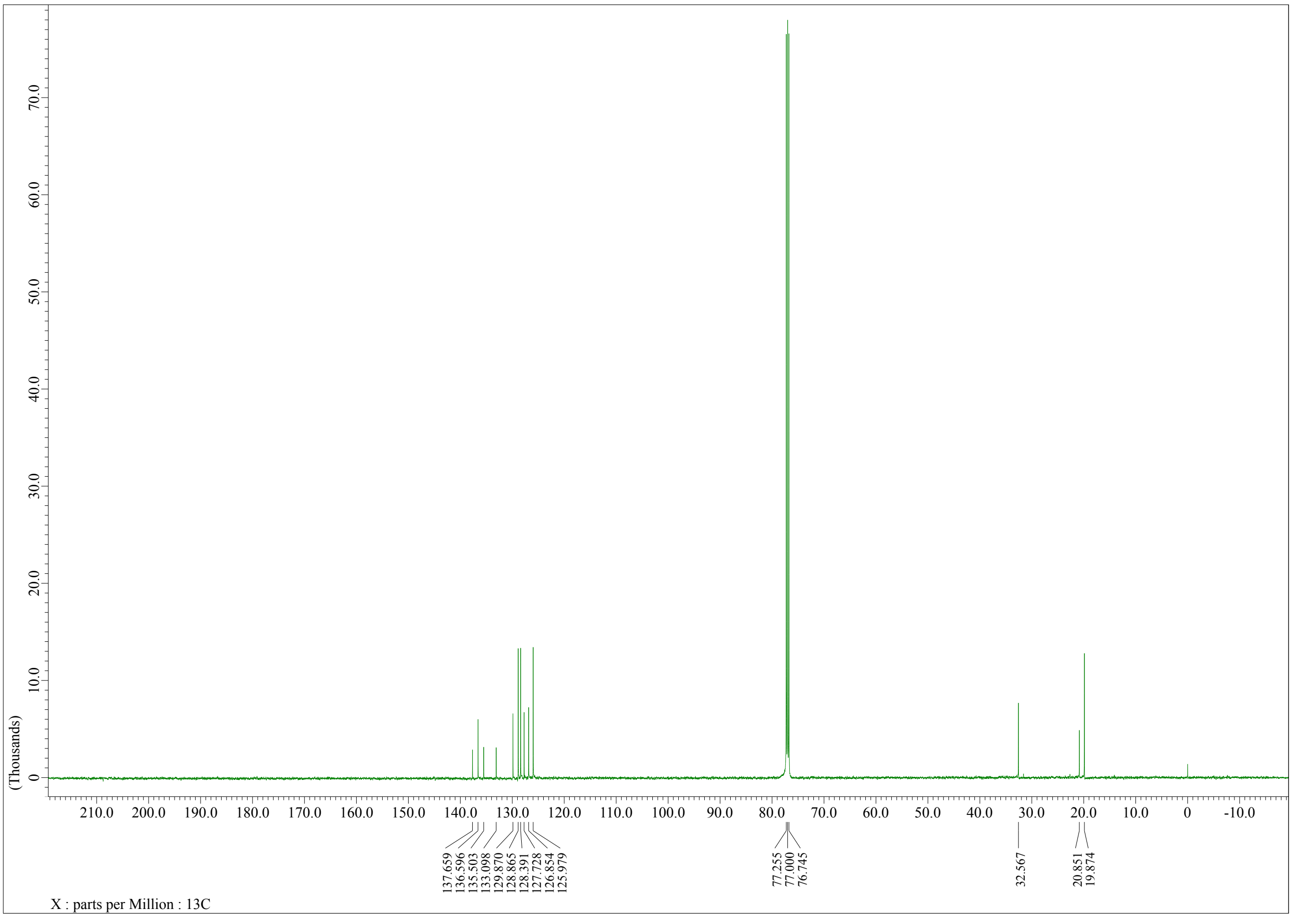
(Thousands)
137.659
136.596
135.503
133.098
129.870
128.865
128.391
127.728
126.854
125.979
77.255
77.000
76.745
32.567
20.851
19.874
X : parts per Million : 13C

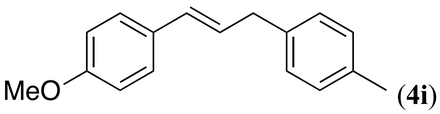
MeO

(4i)

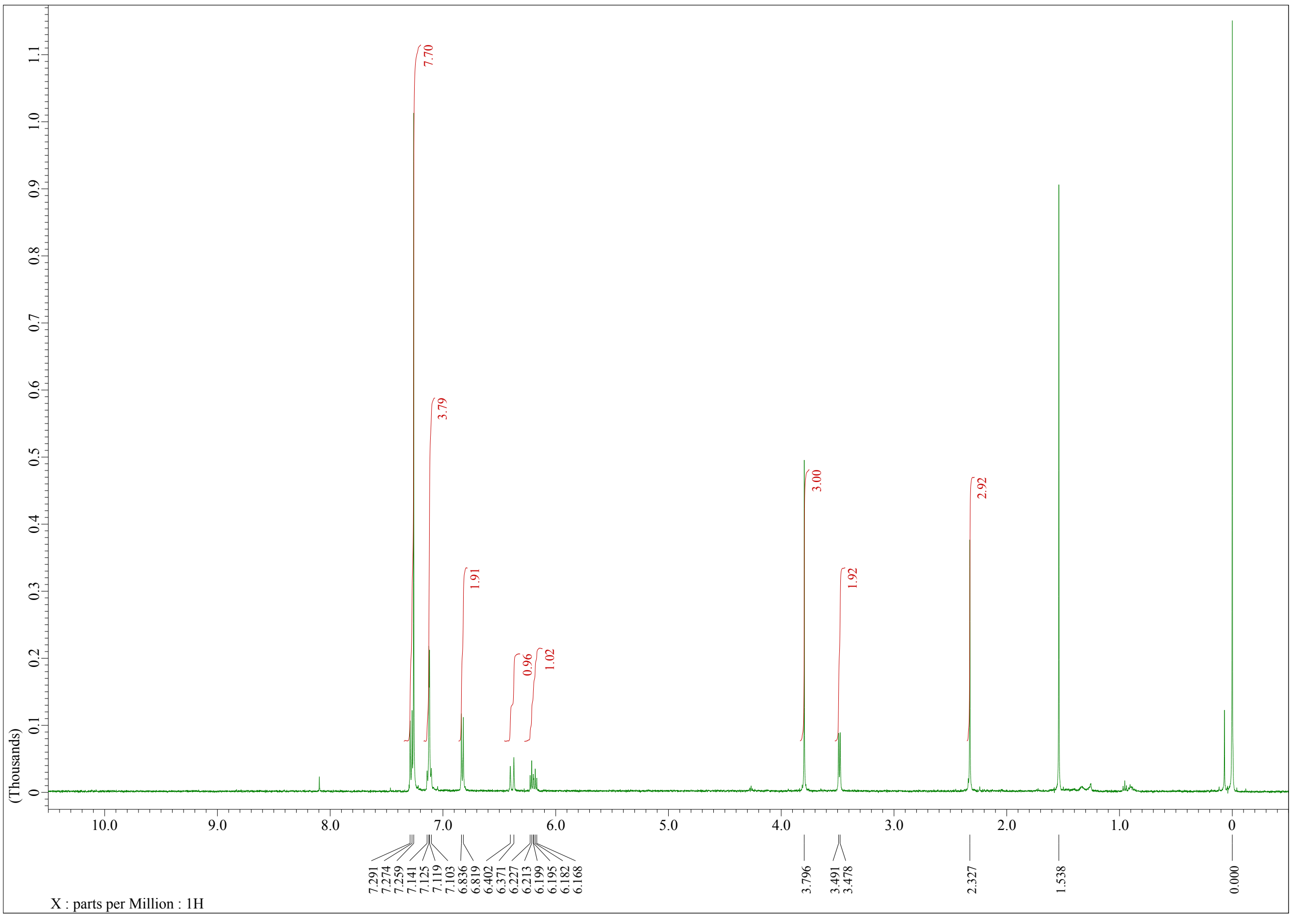
(Thousands)
X : parts per Million : 1H
7.70
3.79
1.91
0.96
1.02
3.00
1.92
2.92
7.291
7.274
7.259
7.141
7.125
7.119
7.103
6.836
6.819
6.402
6.371
6.227
6.213
6.199
6.195
6.182
6.168
3.796
3.491
3.478
2.327
1.538
0.000

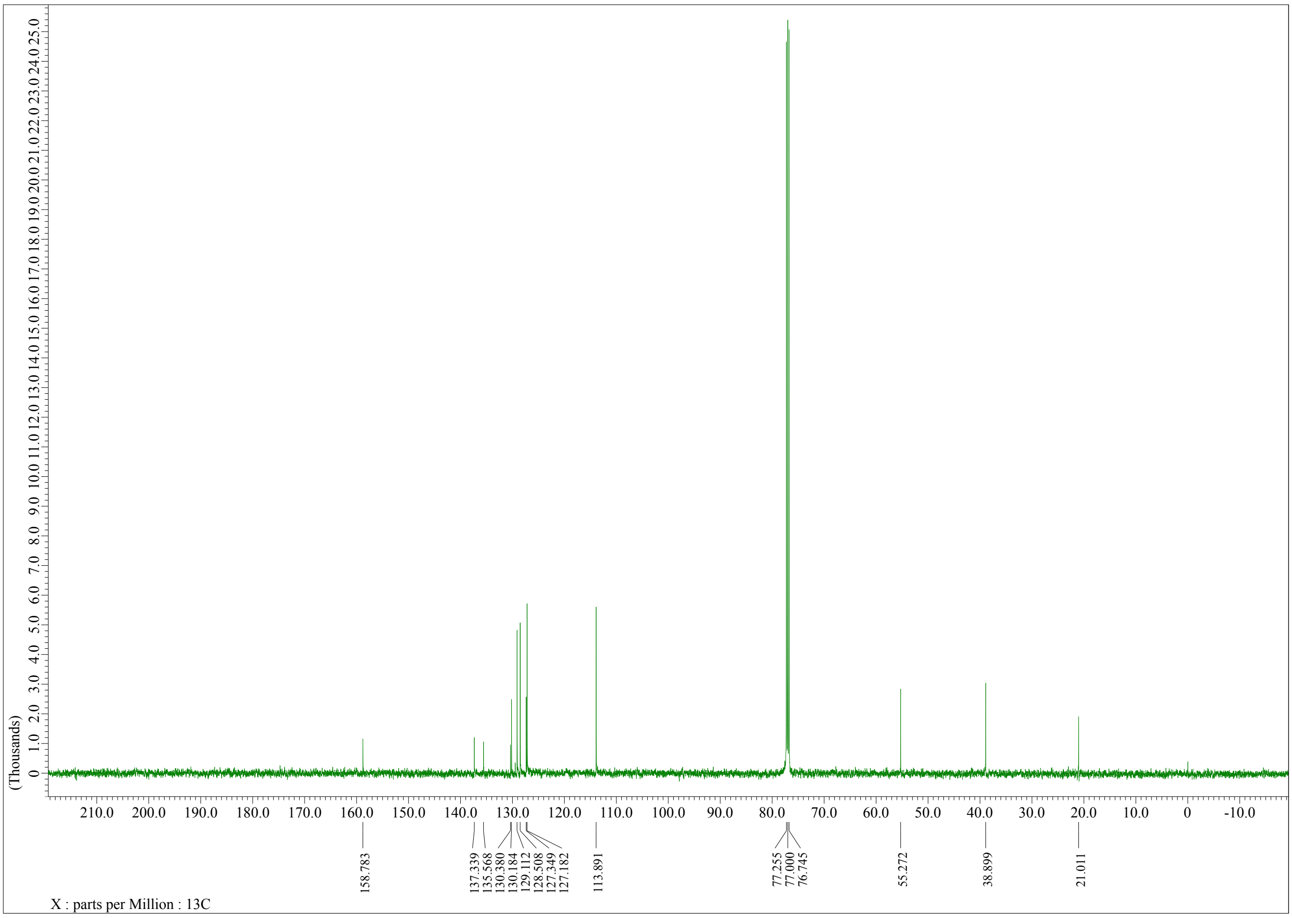
(Thousands)
X : parts per Million : 13C
158.783
137.339
135.568
130.380
130.184
129.112
128.508
127.349
127.182
113.891
77.255
77.000
76.745
55.272
38.899
21.011

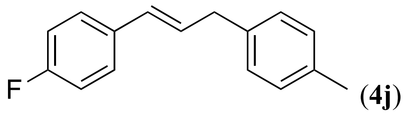
F

(4j)

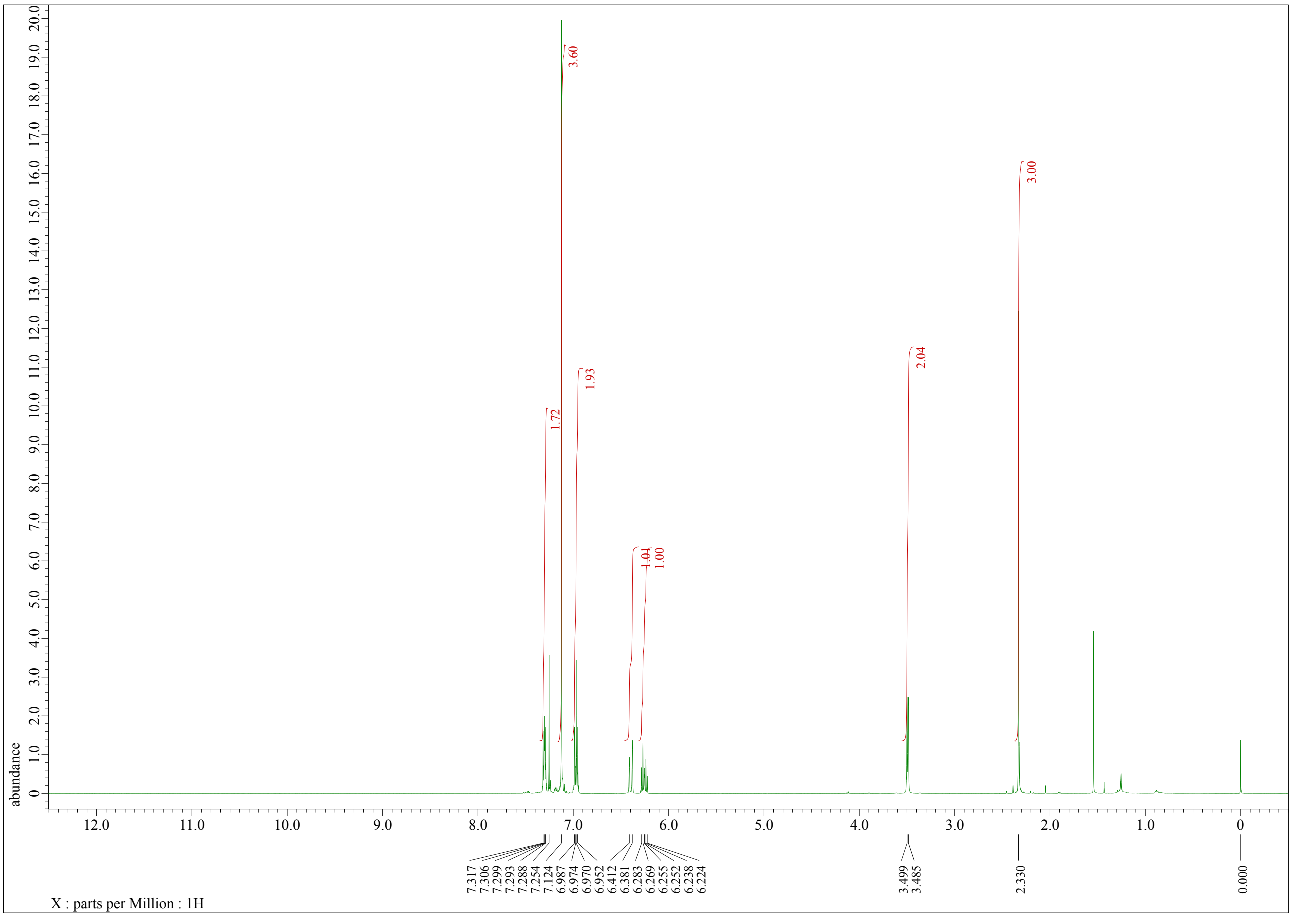
abundance
X : parts per Million : 1H
3.60
1.72
1.93
1.01
1.00
2.04
3.00
7.317
7.306
7.299
7.293
7.288
7.254
7.124
6.987
6.974
6.970
6.952
6.412
6.381
6.283
6.269
6.255
6.252
6.238
6.224
3.499
3.485
2.330
0.000

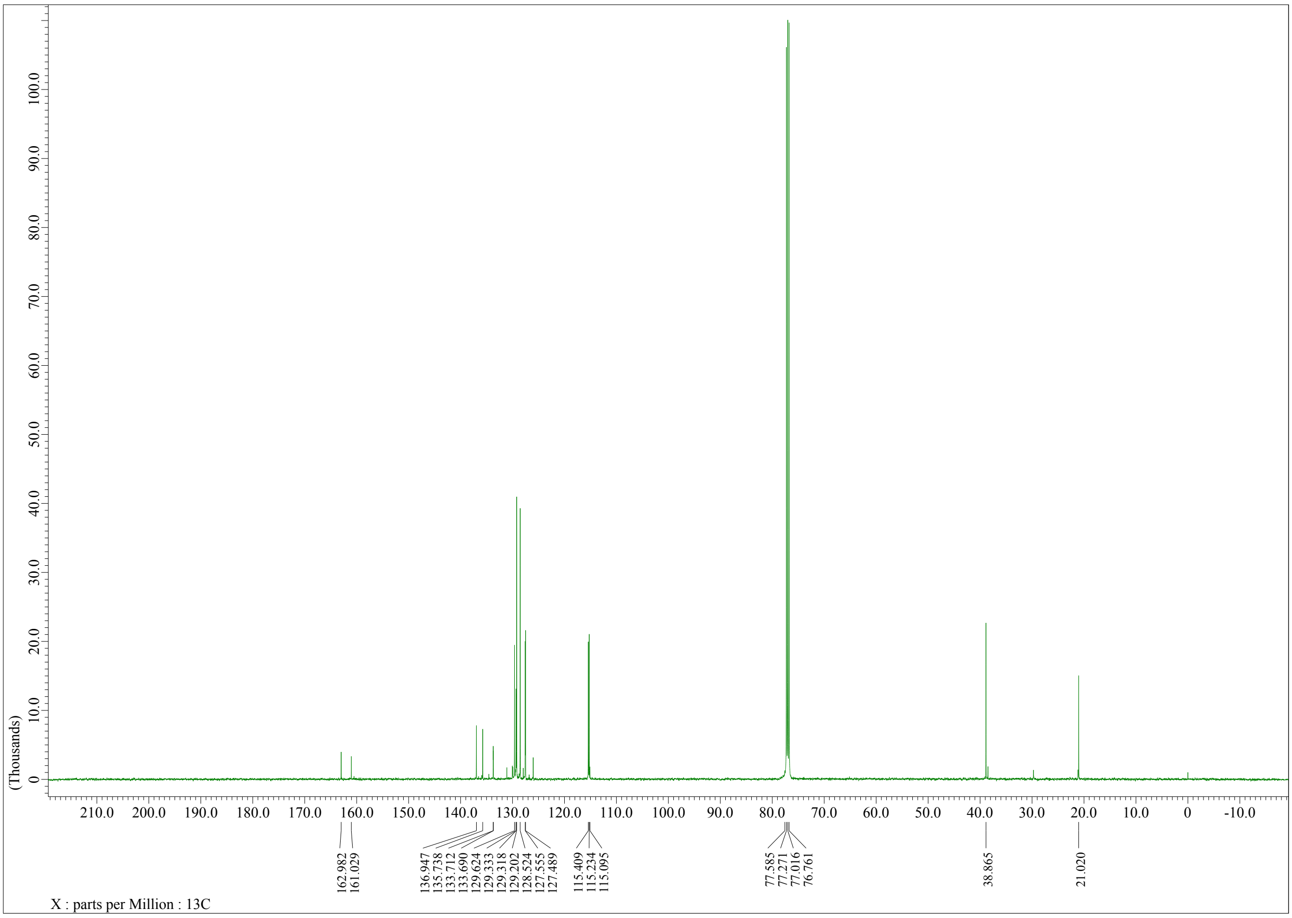
(Thousands)
X : parts per Million : 13C
162.982
161.029
136.947
135.738
133.712
133.690
129.624
129.333
129.318
129.202
128.524
127.555
127.489
115.409
115.234
115.095
77.585
77.271
77.016
76.761
38.865
21.020

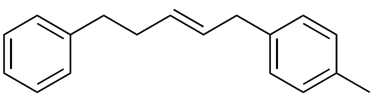
(4k)

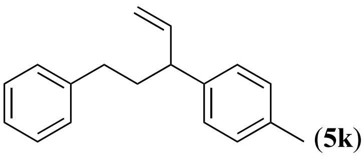
(5k)

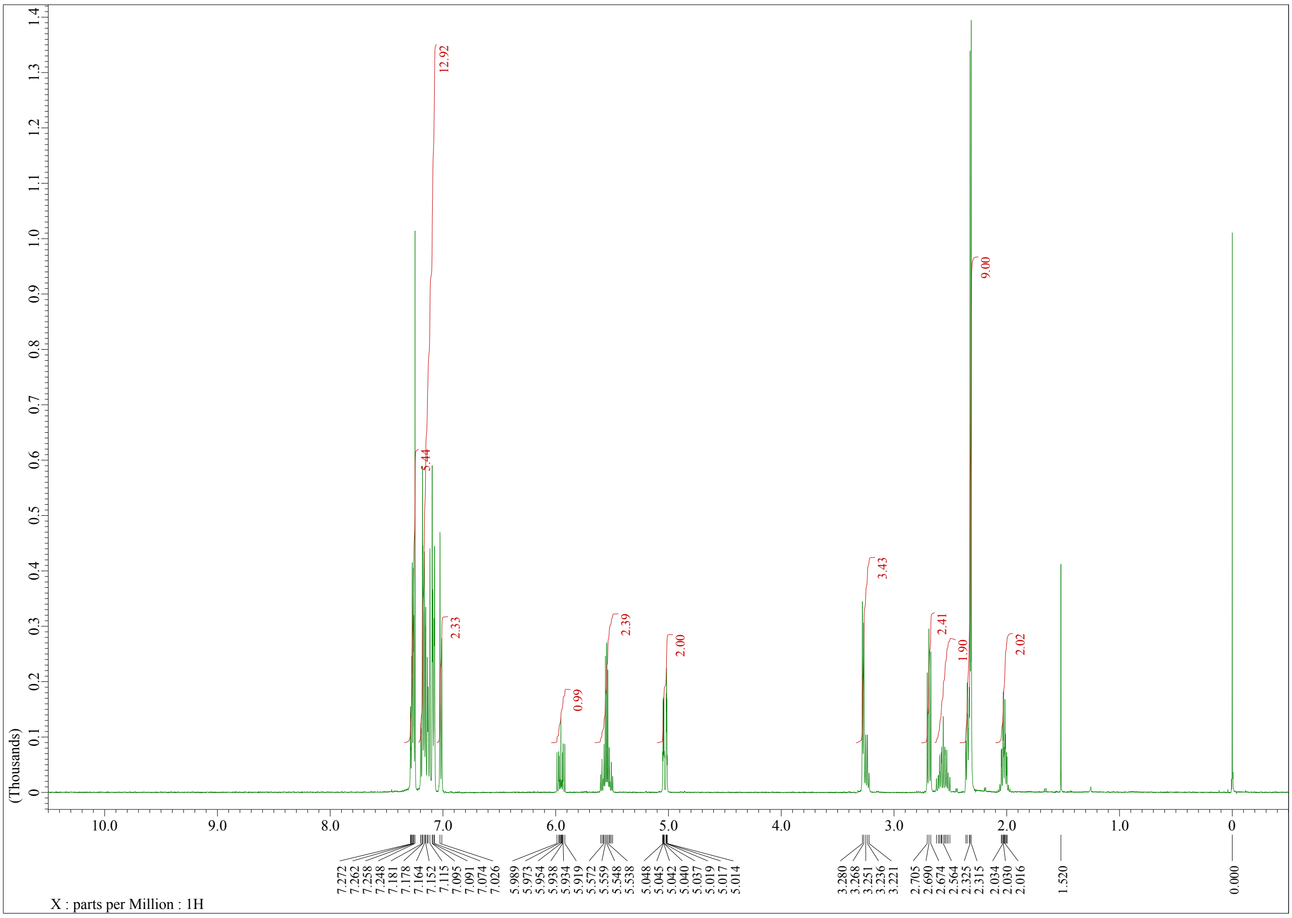
12.92
5.44
2.33
0.99
2.39
2.00
3.43
2.41
1.90
9.00
2.02
7.272
7.262
7.258
7.248
7.181
7.178
7.164
7.152
7.115
7.095
7.091
7.074
7.026
5.989
5.973
5.954
5.938
5.934
5.919
5.572
5.559
5.548
5.538
5.048
5.045
5.042
5.040
5.037
5.019
5.017
5.014
3.280
3.268
3.251
3.236
3.221
2.705
2.690
2.674
2.564
2.325
2.315
2.034
2.030
2.016
1.520
0.000
(Thousands)
X : parts per Million : 1H

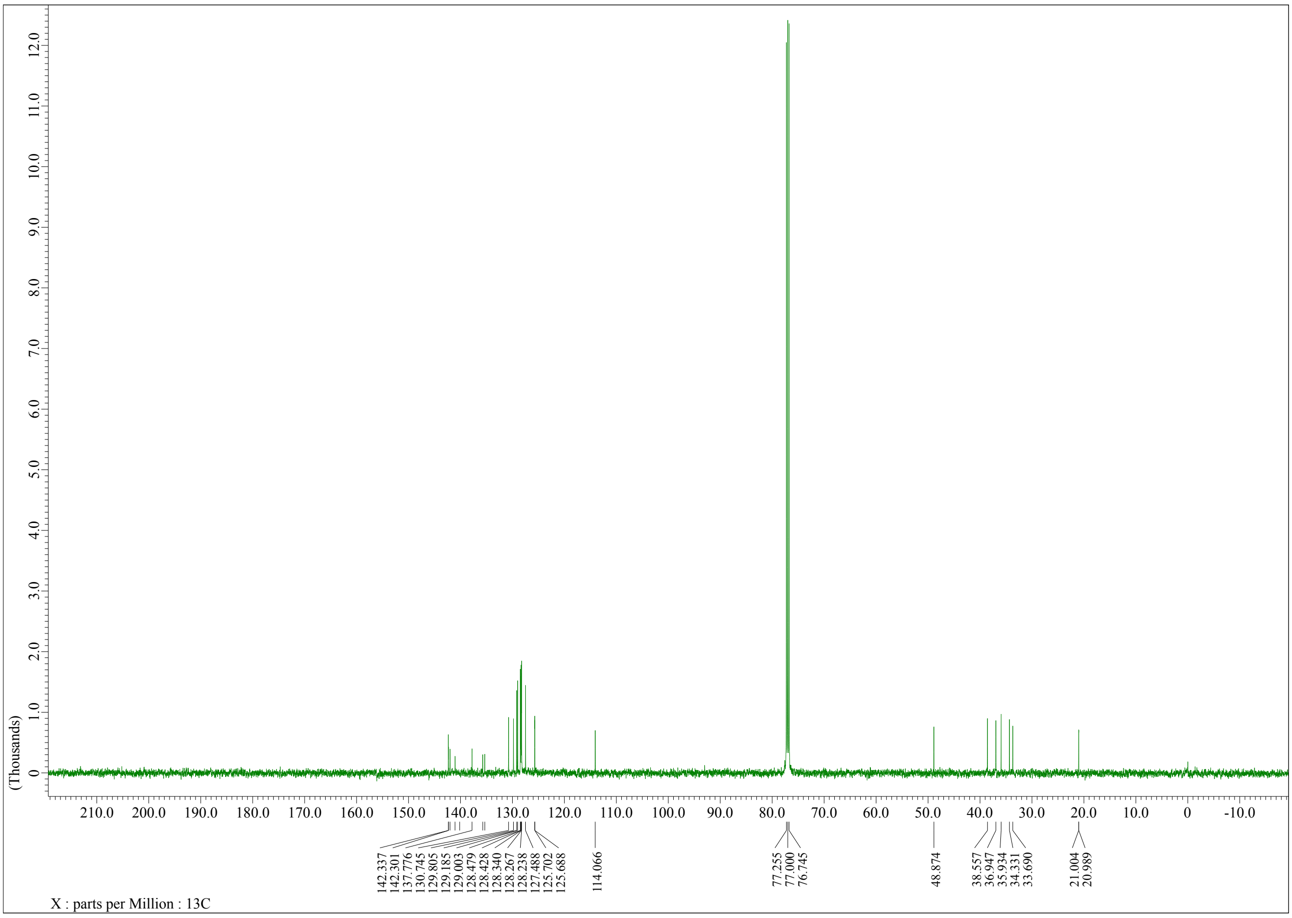
142.337
142.301
137.776
130.745
129.805
129.185
129.003
128.479
128.428
128.340
128.267
128.238
127.488
125.702
125.688
114.066
77.255
77.000
76.745
48.874
38.557
36.947
35.934
34.331
33.690
21.004
20.989
(Thousands)
X : parts per Million : 13C

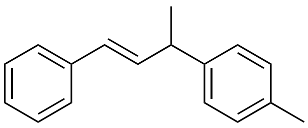

(4l)

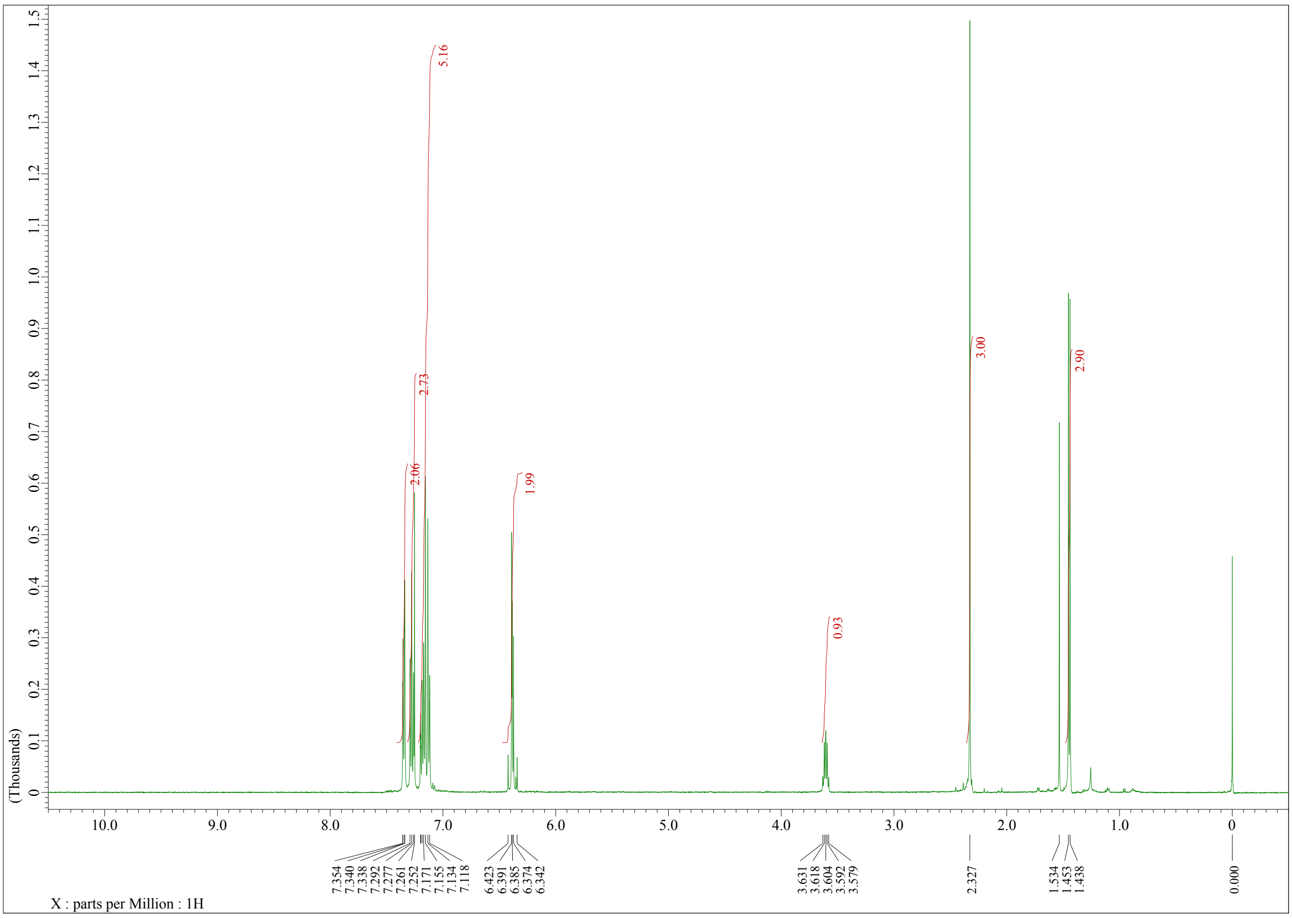

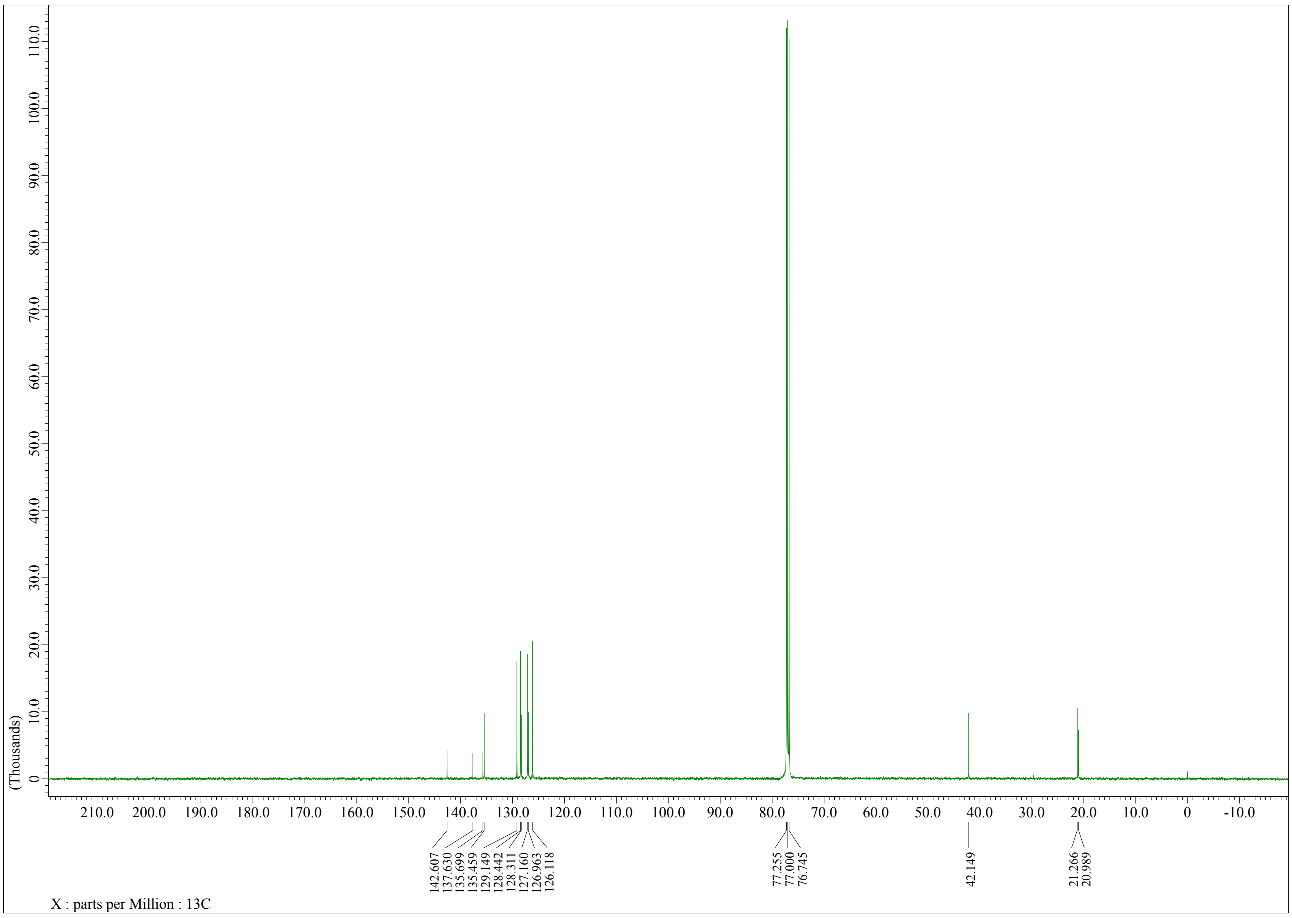

Supplement: Supplementary file 1 [file molecules-24-02296-s001.pdf]
